# Simultaneous Broadband Nonreciprocal Thermal Emission for Transverse Magnetic and Electric Polarizations

**DOI:** 10.1002/nap2.70046

**Published:** 2026-03-20

**Authors:** Junyang Sui, Siyuan Liao, Haifeng Zhang

**Affiliations:** ^1^ College of Electronic and Optical Engineering and the College of Flexible Electronics (Future Technology) Nanjing University of Posts and Telecommunications Nanjing China; ^2^ State Key Laboratory of Millimeter Waves Southeast University Nanjing China

**Keywords:** Berreman mode, broadband nonreciprocal thermal radiation, dual polarizaiton, gradient epsilon‐near‐zero, Weyl semimetals

## Abstract

Objects violating Kirchhoff's law of thermal radiation exhibit unequal absorptivity or emissivity at symmetrical polar angles relative to the normal direction, providing prerequisites for approaching thermodynamic limits in radiative cooling and photon energy conversion, such as the Landsberg limit. Previous studies have demonstrated that broadband nonreciprocal thermal radiation (NTR) can be achieved using magnetized epsilon‐near‐zero (ENZ) materials, such as InAs or Weyl semimetals (WSMs), overcoming the narrowband limitations of most conventional NTR emitters. However, constrained by the inherent properties of magnetized materials, significant broadband NTR is only realized under transverse magnetic mode, fundamentally limits the thermodynamic efficiency under dual‐polarization conditions. This work combines multilayer gradient ENZ WSMs with metasurface geometric construction to achieve, for the first time, significant broadband (8–14 μm) NTR in both transverse magnetic and transverse electric modes. It also exhibits robustness to incident waves directionality. This structure achieves strong nonreciprocity of absorptivity and emissivity under dual‐polarization. The performance is enabled by the geometric asymmetry inducing polarization conversion to overcome polarization mismatch, whereas the magnetized ENZ WSMs fundamentally break Lorentz reciprocity. This research further advances the development of broadband NTR under multipolarization conditions and can be extended to other magnetized metals, III–V semiconductors, magnetized ENZ metamaterials, and metasurfaces.

## 1 Introduction

All matter at nonzero temperatures inevitably emits thermal radiation. Harnessing this radiative energy is of profound importance for diverse applications, including thermal management [1, 2], energy conversion [3], and sensing systems [4, 5]. Rapid progress in nanophotonics has stimulated the emergence of advanced structures and materials, such as metamaterials [6, 7], metasurfaces [8, 9], photonic crystals [10], and two‐dimensional materials [11], which provide flexibly control over the directional, spectral, and polarization characteristics of thermal emission. However, most thermal emission and absorption processes are still fundamentally constrained by Kirchhoff's law, which stipulates that the directional spectral emissivity and absorptivity must be equal [12, 13]. Although this principle traditionally guides conventional thermal emitters design, it also imposes unavoidable energy losses and theoretical efficiency ceilings in energy conversion systems, including photovoltaics and thermophotovoltaics. For example, thermophotovoltaic devices constrained by reciprocity are theoretically capped at 86.8% efficiency, which is notably lower than the nonreciprocal Landsberg limit of 93.3% [14]. As a result, overcoming the constraints of the reciprocity imposed by Kirchhoff's law has become a central pursuit for advancing the efficiency of thermal energy utilization, with profound scientific and technological implications.

Since the 1970s, discussions have existed regarding nonreciprocal emission and absorption [15]. A milestone came in 2014, Zhu and Fan proposed a photonic crystal structure based on magneto‐optical media, theoretically demonstrating nonreciprocal thermal radiation (NTR) in the infrared regime, where the directional spectral emissivity and absorptivity are unequal [13]. Subsequently, the NTR concept gained widespread attention across diverse material systems, including magneto‐optic materials [16, 17, 18, 19, 20], magnetized metals [21, 22], Kerr nonlinear materials [23, 24], and magnetized ENZ metamaterials [25, 26]. However, the latter two often suffer from complex fabrication demands and extremely narrow operational bandwidths. To enhance NTR, many emitter designs incorporate one or more resonances, such as Tamm mode plasmons [27], Fabry‐Pérot cavities [28, 29], or guided‐mode resonances [16, 30]. However, single‐ or multi‐resonance coupling typically enables narrowband nonreciprocal thermal radiation only at the resonant wavelength *λ*, which is not ideal for practical broadband applications in photovoltaics and thermophotovoltaics. By contrast, epsilon‐near‐zero (ENZ) films can support wavelength‐ and angle‐dependent radiation through Berreman modes, and multilayer ENZ films have been shown to provide broadband emission [3, 31, 32]. However, conventional ENZ materials are isotropic and cannot break Lorentz reciprocity, leaving Kirchhoff's law intact. Recently, magnetized ENZ materials combining magneto‐optic effects with ENZ properties, such as conventional magneto‐optic materials n‐InAs, InSb, or novel magnetic Weyl semimetals (WSMs), have offered new opportunities for achieving broadband NTR [33, 34, 35]. Among these, the latter can achieve NTR without an external magnetic field due to their unique topological nontrivial states and inherent time‐reversal symmetry breaking [36]. For instance, Liu et al. [37] proposed a multilayer NTR emitter based on the gradient ENZ magneto‐optical material InAs and experimentally verified broadband nonreciprocal absorption under an external magnetic field. Shi et al. [38] achieved NTR over a broad spectral and angular range without an external magnetic field using multilayer ENZ WSMs. In these studies, NTR across the broad spectral range was confined to the transverse magnetic (TM) mode (the electric field is parallel to the incident plane). This can be attributed to the introduction of nonzero antisymmetric dielectric tensors *ε*_*XZ*_ and *ε*_*ZX*_ by external magnetic fields in magneto‐optical materials such as InAs. The TM mode exhibits nonreciprocity due to coupling with nonzero electric field components (*E*_*X*_ and *E*_*Z*_ are unequal to zero), whereas the transverse electric (TE) mode (the electric field is vertical to the incident plane) retains reciprocity because it possesses only the uncoupled electric field component *E*_*Y*_ [39]. Similarly, the anomalous Hall conductivity induced by nodal momentum separation 2*b* in WSMs leads to *ε*_*XZ*_∝*iσ*_*XZ*_/*ω*, which interacts exclusively with the TM mode, thereby only confining the nonreciprocal effect to TM polarization [38]. The polarization limitation caused by the inherent properties of the material inevitably leads to inherent energy loss under other polarizations, and the theoretical efficiency of energy harvesting is restricted [14]. Recently, achieving TE‐polarized or dual‐polarized NTR has been paid more attention to. For instance, Wu et al. [29] demonstrated strong TE NTR in Weyl semimetal films driven by Fabry–Pérot resonances. Fang et al. [40] proposed silicon‐based nanopore arrays to achieve dual‐polarization NTR via cavity mode excitations. Zhang et al. [41] utilized generalized transverse magneto‐optical effects in grating structures to realize unpolarized broadband NTR. However, these designs predominantly rely on resonant mechanisms, leading to narrowband operation, narrow‐angle range or bad robustness. Therefore, how to achieve NTR over a wide spectral and angle range in multipolarization channels with good robustness is a valuable and challenging task.

In this work, we simultaneously achieve broadband NTR in both TM and TE modes by combining multilayer gradient ENZ WSMs with metasurface geometric construction techniques. Firstly, the Fermi levels are utilized to adjust the *λ* points corresponding to ENZ of the WSMs, making the *λ* points show a gradient shift and cover the 8–14 μm range. Broadband high absorption is achieved by exciting the Berreman mode through the electric field component *E*_*Z*_ of the incident waves. Furthermore, the intrinsic nonreciprocity of the WSMs enables asymmetric absorption/emission. Subsequently, gold (Au) films are attached to the side of the multilayer WSMs, and the entire structure is tilted counterclockwise by 45° around the +*X*‐axis. Simultaneously, the incident and azimuth angles of the incident waves are adjusted to achieve coupling with the induced currents on the gold surfaces. This generates highly asymmetric polarization conversion for positive and negative incident angles. Consequently, the TM mode waves incident at positive and negative angles experience different degrees of attenuation in the electric field components *E*_*X*_ and *E*_*Z*_ coupling with the WSMs, whereas the TE mode waves also acquire coupled electric field components due to polarization conversion. Ultimately, by combining the intrinsic properties of multilayer WSMs with surface plasmon polaritons (SPPs) [42] excited on the inner surface of the gold layer and electromagnetic shielding on the outer surface, a significant broadband NTR phenomenon is achieved. This results in low emission for positive angles and high emission for negative angles in the TM mode and high emission for positive angles and low emission for negative angles in the TE mode. The effects of incident angle, azimuth angle, and inclination angle on the broadband NTR performance are also systematically investigated. This research advances the development of broadband nonreciprocal thermal emitters toward higher efficiency and opens new avenues for multipolarization utilization of radiated energy.

## 2 Metasurface Design and Working Principle Design and Discussion

Figure 1a illustrates a metasurface supporting broadband NTR under multipolarization incidence, exhibiting significant differences in emissivity or absorptivity at identical incident angles on either side of the normal to the incident plane. This metasurface consists of periodically arranged oblique WSMs‐gold units mounted on a monolithic gold substrate. The optical axis of each unit is oriented relative to the global coordinate system *XYZ* by rotating counterclockwise around the +*X*‐axis by a predetermined angle *γ* = 45°. This rotational configuration not only enables polarization conversion but also amplifies the electromagnetic property differences between waves arriving from opposite sides of the normal. Before detailing the numerical optimization, we qualitatively clarify the physical genesis of this geometric topology. The design is evolved to address specific challenges. (i) Gradient multilayering is adopted to align multiple Berreman modes for broadband response; (ii) L‐shaped gold cladding is designed to induce polarization conversion; and (iii) oblique configuration is introduced to form a plasmonic antenna to enhance this conversion efficiency and generate electromagnetic shielding. Based on this physically established topology, the specific geometric parameters are then determined via global optimization. Figure 1b details the meta‐atom structure optimized using the artificial rabbit optimization algorithm (details of artificial rabbit optimization algorithm process are discussed in Supporting Information S1: Section 1). Each meta‐atom consists of an oblique WSMs‐gold structure attached to the gold substrate, where both sides of the multilayer WSMs are covered by gold films colored yellow denoted as G_1_ and G_2_. We define the surface of gold films in contact with air as the outer surface and the surface of gold films in contact with WSMs as the inner surface. Viewed along the ‐*X*‐axis from the side (view 1 in Figure 1b), the regions shaded from dark to light blue represent seven layers of WSMs arranged along the +*Z*‐axis with different Fermi levels (*E*_*F*1_∼*E*_*F*7_) varying from 165 to 92 meV. Their corresponding heights *h*_1_∼*h*_7_ are listed in Table 1. The thickness of G_1_ is *d*_G1_ = 50 nm. The height of the gold substrate is *h*_substrate_ = 50 nm. Viewed along the ‐*Z*‐axis from above (view 2 in Figure 1b), the widths of G_1_, G_2_, and WSMs are *w*_G1_ = 730 nm, *w*_G2_ = 300 nm, and *w*_WSMs_ = 430 nm, respectively. The cross‐sectional lengths of G_2_ and WSMs are *l*_G2_ = *l*_WSMs_ = 1885 nm. Notably, the surface connection between G_1_ and G_2_ at this viewing angle forms an L‐shaped nanoantenna, enhancing polarization conversion performance. View 3 (see Figure 1b) shows another side along the +*X*‐axis, where a single gold film strictly covers the side of the multilayer WSMs. The height *h*_G2_ = 2696 nm corresponds to the combined height of seven WSMs layers. Under specific conditions, SPPs at multiple wavelengths are generated at the contact interfaces between different WSMs layers and the inner surface of the gold films, contributing to absorption.

**FIGURE 1:**
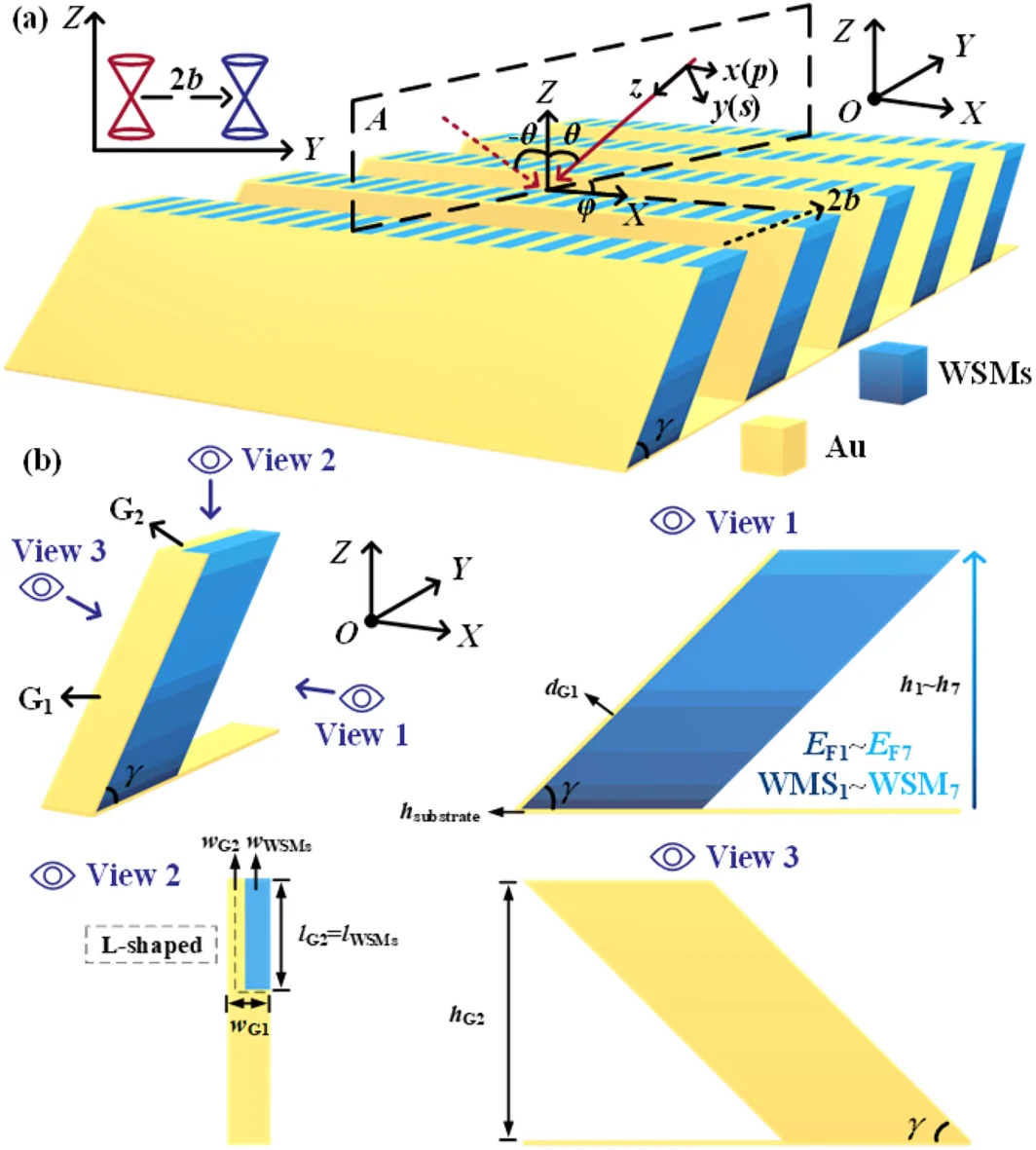
Schematic of the proposed metasurface and details of the meta‐atom structure. (a) Metasurface composed of periodically arranged oblique WSMs‐gold units on a gold substrate. *XYZ* and *xyz* is the global and local coordinate system. Each unit rotated counterclockwise by *γ* = 45° about the +*X*‐axis. *φ* denotes the azimuth angle of plane *A*, where incident angles are +*θ* and −*θ* on either side of the normal. The insect is the electronic band structure of seven‐layer WSMs, where the distance between two Weyl nodes with opposite chirality in momentum space is 2*b*, assumed to be aligned along the +*Y*‐axis. (b) The seven‐layer gradient ENZ WSMs is inclined on a gold substrate with gold films G_1_ and G_2_ on both sides. The Fermi levels of the WSMs increase from bottom to top as EF_1_ to EF_7_. Three observation directions (blue arrows and eye icons) are defined. View 1 is a side view along the −*X*‐axis, View 2 is a top view along the −*Z*‐axis, and View 3 is a side view along the +*X*‐axis.

**TABLE 1:** The Fermi levels and heights of WSMs layers.

Ordering	Layer	Fermi levels *E*_*Fs*_ (meV)	Heights (nm)
1	WSM_1_	165	100
2	WSM_2_	146	150
3	WSM_3_	132	344
4	WSM_4_	120	471
5	WSM_5_	108	174
6	WSM_6_	100	1200
7	WSM_7_	92	257

In WSMs, the momentum separation at the Weyl node (denoted as 2*b*) can be regarded as an axial vector, whose behavior resembles that of an intrinsic magnetic field. When nonzero momentum separation exists, the permittivity tensor of WSMs exhibits off‐diagonal components with opposite signs, leading to the breaking of Lorentz reciprocity [43]. Since the opposite signs of 2*b* in different WSMs layers (e.g., −2*b* for WSM_1_ and +2*b* for WSM_2_) would hinder the realization of high emissivity within the continuous spectrum [44], the 2*b* sign is kept consistent across all WSMs layers in this study. By selecting a specific orientation along the +*Y*‐axis, the permittivity tensor of the Weyl semimetal can be expressed as follows [43]:

(1) $$\varepsilon_{\text{WSMs}}=\left[\begin{array}{ccc} \varepsilon_{d} & 0 & i\varepsilon_{a}\\ 0 & \varepsilon_{d} & 0\\ -i\varepsilon_{a} & 0 & \varepsilon_{d} \end{array}\right],$$

where the components are as follows:

(2) $$\varepsilon_{a}=\frac{be^{2}}{2\pi^{2}\hbar \omega \varepsilon_{0}},$$

(3) $$\begin{array}{l} \varepsilon_{d}=\varepsilon_{b}+i\frac{r_{s}g}{6\text{Re}\left(\Omega \right)}\Omega G\left(\frac{E_{F}\Omega }{2}\right)\\ -\frac{r_{s}g}{6\pi \text{Re}\left(\Omega \right)}\left\{\frac{4}{\Omega }\left[1+\frac{\pi^{2}}{3}\left(\frac{k_{B}T}{E_{F}}\right)\right]+8\Omega \int_{0}^{\xi_{C}}\frac{G\left(E_{F}\xi \right)-G\left(\frac{E_{F}\Omega }{2}\right)}{\Omega^{2}-4\xi^{2}}\xi d\xi \right\} \end{array},$$

here *ε*_*b*_ denotes the background permittivity and *E*_*C*_ represents the cutoff energy, above which the electronic band deviates from linearity. The relation between the Fermi energy *E*_*F*_ and *E*_*C*_ is given by *E*_*C*_ = *ξ*_*C*_ *E*_*F*_. *k*_*B*_ and *ћ* correspond to the Boltzmann constant and the reduced Planck constant. The normalized complex frequency is expressed as *Ω* = *ћ*(*ω* + *iτ* ^−1^)/*E*_*F*_, where *τ* ^−1^is the Drude damping rate. The Fermi distribution function is denoted by *n*(*X*), with *G*(*X*) = *n*(−*X*)−*n*(*X*). The effective fine‐structure constant is defined as *r*_*s*_ = *e*_0_^2^/(4π*ε*_0_ *ћν*_*F*_), where *ν*_*F*_ is the Fermi velocity, and *g* denotes the number of Weyl points. Unless otherwise specified, the parameters are taken as *ε*_*b*_ = 6.2, *ξ*_*C*_ = 3, *τ* = 1000 fs, *g* = 2, *b* = 8.5108 m^−1^, and *ν*_*F*_ = 0.85 × 10^5^ m/s [43].

The permittivity of gold is determined by the Drude model, whose expression is as follows [45]:

(4) $$\varepsilon_{\text{Au}}=\varepsilon_{\infty }-\frac{\omega_{p}^{2}}{\omega \left(\omega +i\Gamma \right)},$$

The parameters are taken as the high‐frequency limit permittivity *ε*_∞_ = 1, the plasma frequency *ω*_*p*_ = 1.32 × 10^16^ rad/s, and the relaxation rate *Γ* = 1.24 × 10^14^ rad/s. When the WSMs rotate about the +*X*‐axis, a conventional right‐handed coordinate system rotation matrix *M*_*X*_ is introduced to rotate the permittivity tensor of the WSMs. At this point, the rotation angle *γ* = −45°, and *M*_*X*_^T^ denotes the transpose matrix:

(5) $$M_{X}=\left[\begin{array}{ccc} 1 & 0 & 0\\ 0 & \cos\left(\gamma \right) & \sin\left(\gamma \right)\\ 0 & -\sin\left(\gamma \right) & \cos\left(\gamma \right) \end{array}\right].$$

The incident angle of the incident waves relative to the normal (+*Z*‐axis) is *θ*, creating an incident plane *A* with the normal. When this plane rotates about the +*Z*‐axis, the angle between plane *A* and the +*X*‐axis is the azimuth angle *φ*, causing the permittivity tensor of the WSMs to rotate again. In this work, *θ* = 60° and *φ* = 26° are selected to generate the broadband NTR phenomenon. The rotation matrix is *M*_*Z*_, and *M*_*Z*_^T^ is its transpose matrix:

(6) $$M_{Z}=\left[\begin{array}{ccc} \cos\left(\varphi \right) & \sin\left(\varphi \right) & 0\\ -\sin\left(\varphi \right) & \cos\left(\varphi \right) & 0\\ 0 & 0 & 1 \end{array}\right].$$

Therefore, the composite rotation matrix is *M*_*R*_ = *M*_*Z*_ *M*_*X*_. The permittivity tensor $\overset{═}{\varepsilon_{\text{WSMs}}}$ of WSMs after rotation is as follows:

(7) $$\overset{═}{\varepsilon_{\text{WSMs}}}=M_{R}\varepsilon_{\text{WSMs}}M_{R}^{T}.$$

It needs to be emphasized that since the diagonal permittivity tensors of *ε*_WSMs_ in Equation (1) remains unchanged, the diagonal tensors persist unchanged after undergoing two rotations. This preserves the ability of WSMs to excite Berreman modes and maintains the constancy of the ENZ *ε*_*d*_ at the *λ* point. What changes are the off‐diagonal components that reflect the nonreciprocal properties.

The solid red line denotes an oblique incident waves at a positive incident angle (+*θ*) with respect to the surface normal within the incident plane, whereas the dashed red line corresponds to oblique incidence at a negative incident angle (−*θ*). In the local coordinate system *xyz*, where the wave vector direction of the incident waves is defined as the +*z*‐axis, the TM mode (*p*‐waves) electric field is set parallel to the +*x*‐axis, whereas the TE mode (*s*‐waves) electric field is set parallel to the +*y*‐axis. Due to the designed metasurface owns (i) WSMs possessing nonreciprocity permittivity tensors, (ii) structural anisotropy introduced by the geometric inclination of the WSMs‐gold unit, and (iii) asymmetric distribution of induced currents on the gold surface, the cross‐polarized reflection coefficients *r*_sp_ and *r*_ps_ exhibit significant asymmetric enhancement/suppression at +*θ* and −*θ*. When linearly polarized plane waves incident on the metasurface, the reflected electric field comprises cross‐polarized and copolarized components. The reflection matrix *R*_lin_ describes the relationship between the incident and reflected electric fields as follows:

(8) $$\left[\begin{array}{l} E_{p}^{r}\\ E_{s}^{r} \end{array}\right]=\left[\begin{array}{cc} r_{\text{pp}} & r_{\text{ps}}\\ r_{\text{sp}} & r_{\text{ss}} \end{array}\right]\left[\begin{array}{l} E_{p}^{i}\\ E_{s}^{i} \end{array}\right]=R_{\text{lin}}\left[\begin{array}{l} E_{p}^{i}\\ E_{s}^{i} \end{array}\right],$$

where $r_{jk}=E_{j}^{r}/E_{k}^{i}$. The first subscript *j* and second subscript *k* correspond to the polarization states of the reflected field and incident field. The validity of the following derivation relies on two key assumptions regarding the energy channels. First, transmission is zero due to the optically thick gold substrate. Second, the metasurface operates predominantly in the subwavelength regime, suppressing higher‐order diffraction. The structural periods along *x*‐axis and *y*‐axis are separately *P*_*x*_ = *w*_G1_ = 730 nm and *P*_*y*_ ≈ 4.3 μm. Although *P*_*y*_ is comparable to the shortest operating wavelength, the diffraction channels are strictly constrained. According to the universal modal radiation laws [46, 47], this subwavelength condition (*P* < *λ*/(sin*θ*_*r*_−sin*θ*_*i*_)) ensures that all higher‐order diffraction harmonics are evanescent. Consequently, energy interaction is dominated solely by the zeroth‐order specular reflection (detailed analysis shows in Supporting Information S1: Section 2). Therefore, The directional spectral absorptivity *α*(*λ*,*θ*,*φ*) and emissivity *e*(*λ*,*θ*,*φ*) can be expressed as functions of the directional spectral reflectivity *R*(*λ*,*θ*,*φ*) [16, 17]

(9) $$\alpha \left(\lambda ,\theta ,\varphi \right)=1-R_{kk}\left(\lambda ,\theta ,\varphi \right)-R_{jk}\left(\lambda ,\theta ,\varphi \right),$$

(10) $$e\left(\lambda ,\theta ,\varphi \right)=1-R_{kk}\left(\lambda ,-\theta ,\varphi \right)-R_{jk}\left(\lambda ,-\theta ,\varphi \right).$$

In conventional reciprocal media, Lorentz reciprocity dictates *R*(*λ*,*θ*,*φ*) = *R*(*λ*,−*θ*,*φ*), leading to the conventional Kirchhoff's law, where *α*(*λ*,*θ*,*φ*) = *e*(*λ*,*θ*,*φ*). However, in magneto‐optical systems, such as the WSMs‐based metasurface proposed here, the permittivity tensor is non‐Hermitian and breaks Lorentz reciprocity. This results in asymmetric reflection, that is, *R*(*λ*,*θ*,*φ*)≠*R*(*λ*,−*θ*,*φ*). To satisfy the second law of thermodynamics, the system must adhere to the generalized Kirchhoff's law, which relates emissivity to the absorptivity of the time‐reversed process. For a detailed derivation of the absorptivity and emissivity based on the NTR and the principle of energy conservation, refer to Supporting Information S1: Section 3 [13, 48]. From this, the following relationship holds:

(11) e(λ,θ,φ)=α(λ,−θ,φ).

Based on the above conditions, the expression for the average emissivity *ē*(*λ*,*θ*,*φ*) is given by [49].

(12) $$\bar{e}\left(\lambda ,\theta ,\varphi \right)=\frac{\int_{\lambda_{L}}^{\lambda_{H}}I_{\text{BB}}\left(\lambda \right)e\left(\lambda ,\theta ,\varphi \right)d\lambda }{\int_{\lambda_{L}}^{\lambda_{H}}I_{\text{BB}}\left(\lambda \right)d\lambda },$$

here *λ*_*L*_ and *λ*_*H*_ denote the lower and upper bounds of the *λ* range. *I*_BB_(*λ*) represents the spectral intensity of blackbody radiation at *λ*. Additionally, nonreciprocity is defined as the absolute value of the difference between the directional spectral emissivity and absorptivity, namely [50].

(13) η(λ,θ,φ)=|e(λ,θ,φ)−α(λ,θ,φ)|.

This work employs the finite element method (FEM) to calculate the reflection coefficient spectra of the metasurface under varying *θ*, *φ*, and *γ*, simultaneously determining the relationships between absorptivity, emissivity, and nonreciprocity [51, 52].

## 3 Analysis and Discussion of Performances

The fundamental difference between reciprocal and nonreciprocal absorbers in their *λ* and *θ* responses is illustrated in Figure 2a. For conventional reciprocal systems, the absorptivity and emissivity strictly adhere to Kirchhoff's radiation law, meaning *α*(*λ*,*θ*,*φ*) = *α*(*λ*,−*θ*,*φ*) under the conditions of any *λ*, *θ,* and *φ*. This implies that their absorptivity and emissivity spectra are perfectly coincident, precluding directional selectivity. In nonreciprocal systems incorporating nonreciprocity materials, this symmetry is broken. Techniques, such as guided‐mode resonance, enable narrowband absorptivity peaks with significant differences between +*θ* and −*θ* in the TM mode [16, 27, 28, 29, 30], that is, *α*(*λ*,*θ*,*φ*)≠*α*(*λ*,−*θ*,*φ*). Gradient anisotropic ENZ materials can satisfy broadband nonreciprocal absorption. However, for the TE mode, due to the absence of magneto‐optic coupling [38, 39], it retains its reciprocal properties. This characteristic limits the theoretical constraints of breaking reciprocity in energy conversion under multipolarization conditions. Based on the designed metasurface of the oblique WSMs‐gold unit cell periodic array in Figure 1, the significant broadband NTR performance under multipolarization conditions is achieved. According to Equation (11), *e*_TM_(60°) = *α*_TM_(−60°) and *e*_TE_(60°) = *α*_TE_(−60°). Figure 2b,e separately display the absorptivity and emissivity spectra at *θ* = 60° for TM and TE modes, indicating substantial breaking of reciprocity. The average emissivity in the TM mode at +*θ* and −*θ* is *ē*_TM_(60°) = 0.236 and *ē*_TM_(−60°) = 0.803, with a difference of 0.567. In the TE mode, the values are *ē*_TE_(60°) = 0.746 and *ē*_TE_(−60°) = 0.187, owning a difference of 0.559. These results, calculated from Equation (12), demonstrate the pronounced directional emission selectivity. The blue triangles in Figure 2b,e mark the wavelength points corresponding to the ENZ of the multilayer WSMs, which excite the Berreman mode to ensure absorption in a broad spectrum. It is crucial to distinguish between asymmetric directional emission and true NTR. As demonstrated in recent studies involving geometrically asymmetric structures composed of isotropic materials [8, 53], significant directional asymmetry (*e*(*λ*,*θ*,*φ*)≠*e*(*λ*,−*θ*,*φ*)) can be achieved. However, these systems are fundamentally reciprocal, meaning they strictly adhere to Kirchhoff's law (*e*(*λ*,*θ*,*φ*) = *α*(*λ*,*θ*,*φ*)). In contrast, the design proposed here incorporates magnetized WSMs. The geometric inclination serves primarily as a polarization converter to facilitate mode coupling, whereas the off‐diagonal permittivity of the WSMs fundamentally breaks Lorentz reciprocity. This leads to the violation of the conventional Kirchhoff's law (*e*(*λ*,*θ*,*φ*)≠*α*(*λ*,*θ*,*φ*)), realizing true nonreciprocity rather than mere directional asymmetry. As shown in Figure 2c,f, in the TM mode, the cross‐polarized reflectivity *R*_sp_(60°) and *R*_sp_(−60°) exhibit distinct spectral separation at +*θ* and −*θ*, with *R*_sp_(60°) exceeding *R*_sp_(−60°) across a broad spectrum. In contrast, in the TE mode, the cross‐polarized reflectivity at +*θ* and −*θ* exhibits the broadband phenomenon of *R*_ps_(60°) < *R*_ps_(−60°). These principles will be explained in detail below. Figure 2d,g show the variation curves of nonreciprocity. The difference between absorptivity and emissivity at the same *θ* in TM and TE modes is consistently greater than 0.35, with peak values reaching |*η*_TM_(60°) = 0.696| and |*η*_TE_(60°) = 0.732|, respectively, indicating a pronounced NTR phenomenon. This represents a substantial improvement over |*η*_TM_(60°) = 0.147| reported in Ref. [35] and |*η*_TM_(60°) = 0.3| in Ref. [37].

**FIGURE 2:**
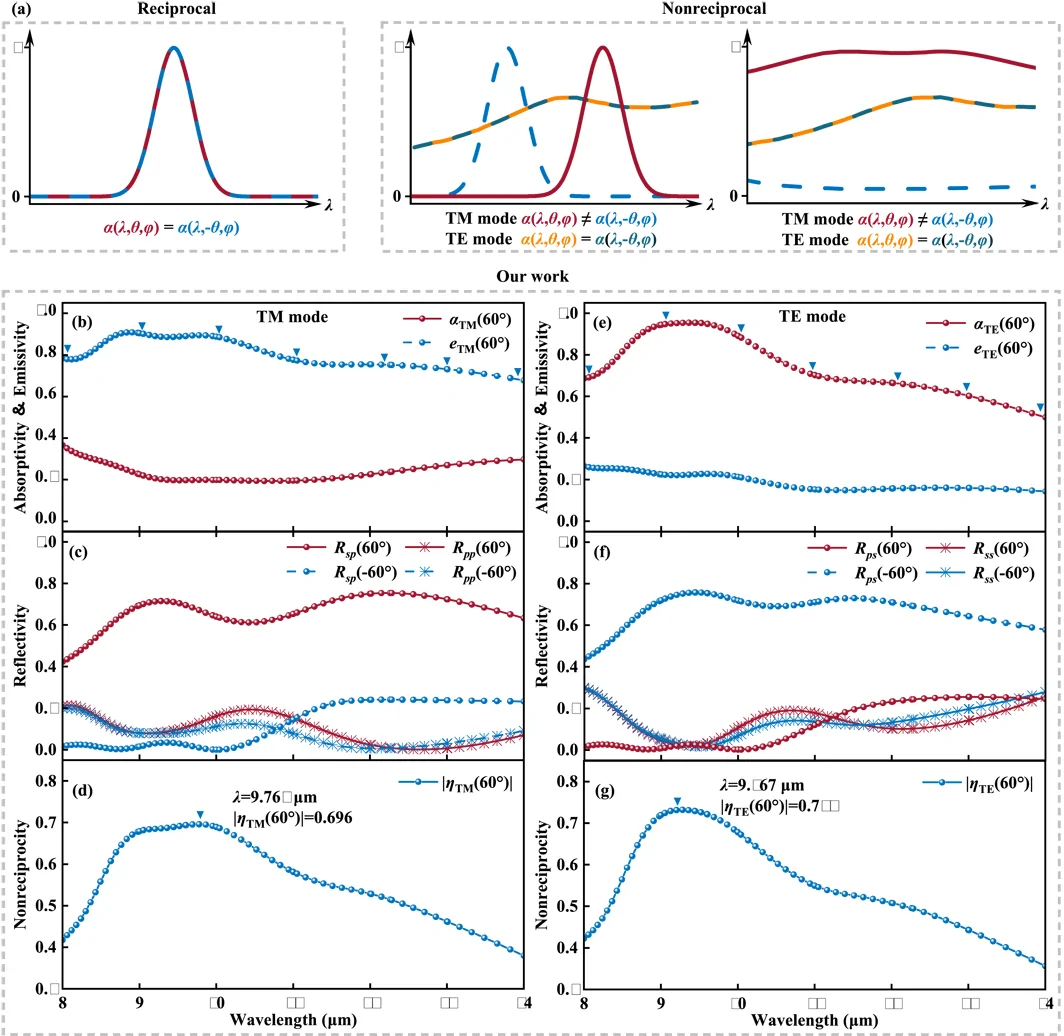
Schematic of broadband NTR in TM and TE modes. (a) Comparative absorptivity spectra of reciprocal (left) versus nonreciprocal (right two subplots) absorbers. Electromagnetic properties spectra are calculated for the metasurface TM mode (b–d) and TE mode (e–g). (b), (e): Absorptivity and emissivity spectra of the metasurface at *θ* = 60° show distinct separation. Corresponding resonance absorptivity/emissivity peaks for each WSMs layer are marked with blue triangles. (c), (f): Copolarized and cross‐polarized reflectivity of the metasurface at *θ* = 60° and −60°. (d), (g): Nonreciprocity of the metasurface at *θ* = 60°, with blue triangles indicating maximum values and corresponding wavelengths.

To explain the role of multilayer WSMs in forming broadband NTR, as shown in Figure 3a, consider the general case where seven layers of WSMs with identical thickness (150 nm) are stacked on a gold substrate without rotation (*γ* = 0°). Fermi levels from the bottom layer upward are taken from Table 1. The gradient blue solid line from dark to light in Figure 3b displays the real part values of *ε*_*d*_, which are close to zero at specific *λ* points of 8.05, 9.08, 10.01, 10.97, 12.1, 12.98, and 13.97 μm, respectively, covering a broad spectral range of 8–14 μm. These values increase as the Fermi levels decrease. ENZ WSMs films exhibit superior performance in electromagnetic energy localization, attributed to satisfying the boundary condition *E*_ENZ_ = *E*_*i*_ *ε*_*i*_/*ε*_*d*_→∞ (where *E*_*i*_ and *ε*_*i*_ represent the electric field and permittivity of adjacent layers). Furthermore, at specific *θ*, the lossy ENZ WSMs films can couple with free space light, exhibiting high absorption of localized electromagnetic energy. The corresponding *λ* can be estimated via the peak of the energy loss function Im(−1/*ε*_*d*_) (dashed line in Figure 3b) [32]. This behavior aligns with the Berreman mode (observed near the longitudinal optical phonon polariton resonance in materials exhibiting phonon absorption) [32]. Alternative interpretations suggest this phenomenon as a leaky TM mode resonant electromagnetic mode (i.e., Brewster mode) excited when in contact with lossy ENZ interfaces such as metals [38, 54]. Due to *ε*_*d*_ crossing zero at specific *λ* values, this process significantly enhances magneto‐optical intensity, quantifiable by the Voigt parameter *ζ* = |*ε*_*a*_|/|*ε*_*d*_|, as shown in Figure 3c. As expected, a peak is observed near the ENZ *λ*. The large ratio indicates the strong intrinsic nonreciprocal nature of WSMs, outperforming InAs with *ζ* less than 3.6 [55].

**FIGURE 3:**
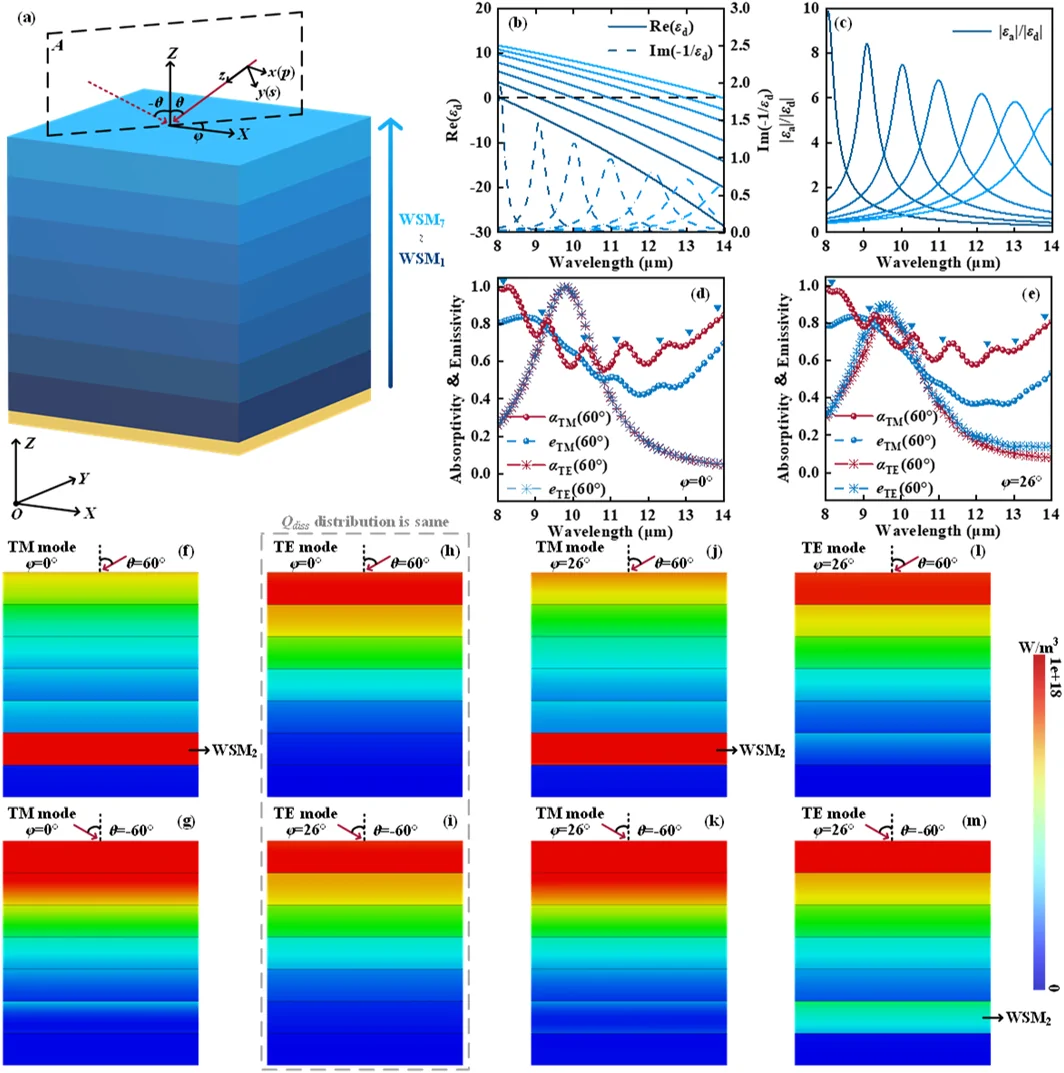
Analysis of the role of WSMs layers in forming broadband NTR effects under general case. (a) Seven layers of gradient ENZ WSMs are vertically placed (*γ* = 0°) on a gold substrate. All layers have the same thickness of 150 nm. The Fermi levels *E*_*F*1_∼*E*_*F*7_ along the +*Z*‐axis from bottom to top are assigned values according to Table 1 *φ* denotes the azimuth angle of plane *A*, with incident waves angles +*θ* and −*θ* on either side of the normal. (b) Permittivity tensor curves for the seven‐layer WSMs. The real part Re(*ε*_*d*_) of *ε*_*d*_ for WSMs films at different Fermi levels (solid lines). The dashed lines indicate *λ* of the Berreman mode peak obtained via the energy dissipation function lm(−1/*ε*_*d*_). (c) Ratio of the permittivity tensors |*ε*_*a*_| and |*ε*_*d*_|. The peak affected by the ENZ corresponds to the *λ* where WSMs exhibit maximum nonreciprocity. (d) Absorptivity and emissivity spectra for TM and TE modes at *θ* = 60° and *φ* = 0°. The TM mode spectra are separated, whereas the TE mode spectra remain overlapping. Blue triangles mark the *λ* positions where WSMs exhibit ENZ. (e) Absorptivity and emissivity spectra of TM and TE modes at *θ* = 60° and *φ* = 26°, showing separated spectral lines. At the selected *λ* = 9.08 μm, the spatial distribution of *Q*_diss_ for incident waves at *θ* = 60° and −60° in TM and TE modes, respectively, corresponding to (f–i) *φ* = 0° and (j–m) *φ* = 26°.

Figure 3d shows the absorptivity and emissivity spectra for TM and TE modes at *θ* = 60° and *φ* = 0°. In the TM mode, due to the intrinsic nonreciprocal interaction of the WSMs, the absorptivity spectrum exhibits an enhancement modulation at the WSMs ENZ *λ* points relative to the emissivity spectrum. The blue triangles mark high absorption corresponding to the Berreman mode, where the WSMs layers are excited by the electric field *E*_*Z*_ component of the incident waves. In contrast, the absorptivity and emissivity spectra for the TE mode cannot be separated. This is attributed to the absence of the electric field *E*_*X*_ and *E*_*Z*_ components, preventing interaction with the anomalous Hall conductivity introduced by the momentum separation of the Weyl node in the WSMs, thereby losing nonreciprocity [38]. The TE mode absorptivity originates solely from the imaginary part of the WSMs permittivity. When the incident plane *A* rotates around the +*Z*‐axis to yield an azimuth angle *φ* = 26°, Figure 3e shows the absorptivity and emissivity for both TM and TE modes. In addition to a larger nonreciprocal separation occurring in the TM mode, the absorptivity and emissivity spectra in the TE mode also exhibit separation. The presence of the *φ* endows the incident TE mode waves with the *E*_*X*_ component. Simultaneously, influenced by the WSMs own optical rotation properties, a portion of the incident waves undergoes polarization conversion during contact with the layered structure, thereby acquiring the *E*_*Z*_ component needed by the Berreman mode of the ENZ WSMs. The presence of both *E*_*X*_ and *E*_*Z*_ components can couple with the antisymmetric permittivity tensor of the WSMs, leading to asymmetric absorption at +*θ* and −*θ*. Conversely, for TM mode incident waves, polarization conversion can weaken the *E*_*X*_ and *E*_*Z*_ components at +*θ* and −*θ* to varying degrees, thereby widening the nonreciprocal separation of spectral lines. This intriguing discovery inspires us to enhance the separation of absorptivity and emissivity for TM and TE modes by (i) increasing the polarization conversion of the structure and (ii) amplifying the polarization conversion asymmetry at +*θ* and −*θ*. These approaches enable significant broadband NTR across multiple polarization states.

To gain a clearer understanding of the physical mechanism behind the peak of *e*(*λ*, *θ*, *φ*) at a specific *λ*, the electromagnetic power dissipation density (denoted as *Q*_diss_) within multilayer films is visualized at a given *λ*. Here, *Q*_diss_(*x*,*y*,*z*)|_*λ*,*θ*,*φ*_ represents the absorption/power dissipation density at specific *λ*, *θ*, and *φ*, which relates simply to the absorptivity:

(14) $$\alpha \left(\lambda ,\theta ,\varphi \right)=\frac{\int_{V\left(x,y,z\right)}Q_{\text{diss}}\left(x,y,z\right){|}_{\lambda ,\theta ,\varphi }dV}{P_{\text{incident}}{|}_{\lambda ,\theta ,\varphi }}.$$

According to Equations (11) and (14), the *Q*_diss_(*x*,*y*,*z*)|_*λ*,*θ*,*φ*_ distribution can be equivalently interpreted as the *e*(*λ*,−*θ*,*φ*) observed at −*θ*. *λ* is selected as 9.08 μm. Figure 3f–i show the *Q*_diss_ distributions at *φ* = 0° for *θ* = 60° and *θ* = −60°. Attributed to Berreman mode excitation, the *Q*_diss_ at *θ* = 60° in the TM mode is predominantly concentrated within the WSM_2_ layer, indicating that most electromagnetic energy is absorbed within this film layer. This aligns with the ENZ correspondence of Re(*ε*_*d*_) at *λ* = 9.08 μm under the *E*_*F*2_ = 146 meV in Figure 3b. Due to intrinsic nonreciprocity, at *θ* = −60°, the *Q*_diss_ intensity in the corresponding WSMs layer decreases, exhibiting a typical distribution localized at the top layer, a pattern characteristic of *Q*_diss_ arising solely from the imaginary part of the permittivity. This scenario results in reduced absorption efficiency. In the TE mode, no distinction exists in the *Q*_diss_ distribution at +*θ* and −*θ* as no corresponding electric field component excites the intrinsic nonreciprocity of the WSMs. However, at *φ* = 26°, as shown in Figure 3j–m, besides the significant *θ* dependent variation of *Q*_diss_ in the WSM_2_ layer under TM mode, the distribution of *Q*_diss_ at *θ* = 60° and −60° also exhibits distinct differences under TE mode, reflecting the separation of absorptivity spectra. Specifically, the *E*_*X*_ and *E*_*Z*_ components generated by polarization conversion couple with the antisymmetric permittivity tensor of the WSMs. Meanwhile at *θ* = −60°, the generated *E*_*Z*_ component excites the Berreman mode in the WSM_2_ layer, leading to an increase in *Q*_diss_. This corresponds to the overall higher *e*_TE_(60°) value compared to *α*_TE_(60°) in Figure 3e. The discussion on the arrangement of the WSMs layers can be found in Supporting Information S1: Section 4.

The following explains how to enhance the polarization conversion and increase the asymmetry of the structure by utilizing metasurface geometric construction techniques and the surface induced current of gold films. First, we discuss the case of incident waves (represented by red solid lines) in TM and TE modes under conditions of *θ* = 60° and *φ* = 26°. Figure 4a shows the oblique WSMs‐gold unit constructed in Figure 1b, with top view 2 and rear view 4. Projecting the electric field vectors of the incident TM and TE modes in the local coordinate system *xyz* onto the global coordinate system *XYZ*. A pair of orthogonal axes *u*‐axis and *v*‐axis can be defined along the direction of the projected electric field. Figure 4b displays the cross‐polarized and copolarized reflection coefficients for the TM mode. The high cross‐polarized reflection coefficient *r*_sp_(60°) indicates significant polarization conversion occurring when the TM mode incident waves at *θ* = 60°. This is explained by the induced current on the gold films surfaces shown in Figure 4f. Under the incident TM mode waves at *λ* = 9.08 μm, the current on the upper surface (view 2) intensifies along the lower arm of the L‐shaped nanoantenna and the slope of gold film G_1_, running parallel to the *X*‐axis. *J*_*S*_ = *σ*_gold_ *E*_*S*_ (where *J*_*S*_, *σ*_gold_, and *E*_*S*_ denote the current density, electrical conductivity of gold, and tangential electric field component) provides an electric field in the direction of the surface current. Conversely, the induced current on the gold substrate, parallel to the *Y*‐axis, provides an electric field in that direction. These two electric fields decompose into components parallel to the *u*‐ and *v*‐axes. Their coupling generates an electric field along the *v*‐axis. Furthermore, the inner surface of the inclined gold film G_1_, together with the gold substrate, interferes with the incident waves, affecting the *E*_*Z*_ component. These phenomena indicate that the electric field of the reflected waves is deflected toward the *y*‐axis direction (TE mode) in the *xyz*. The same principle applies at *λ* = 10.97 μm, though the current intensity on the top gold surface and gold substrate is weaker, resulting in a smaller polarization conversion. This is embodied in Figure 4b as *r*_sp_(9.08 μm, 60°) > *r*_sp_(10.97 μm, 60°). Since the *E*_*Z*_ electric field component of TM mode is significantly reduced due to polarization conversion, the excitation of the Berreman mode is weakened. Consequently, the distribution intensity of *Q*_diss_ in the corresponding WSMs layer under the view 4 in Figure 4g is markedly diminished, reducing incident waves absorption.

**FIGURE 4:**
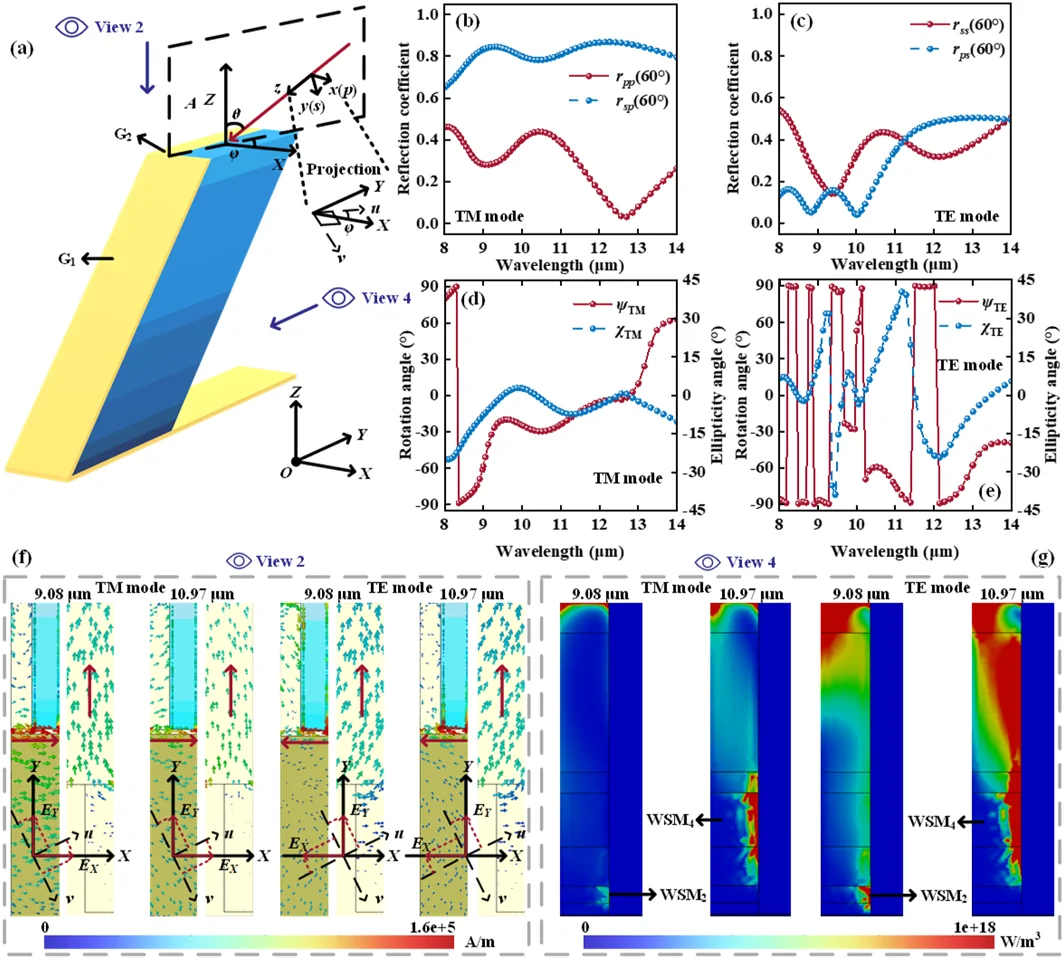
Performance analysis of the oblique WSMs‐gold unit at *θ* = 60° and *φ* = 26°. (a) Designed oblique WSMs‐gold unit. Within the incident plane at *φ*, the incident waves (red solid line) strikes at *θ* = 60°. The local coordinate system *xyz* is projected onto the global coordinate system *XYZ*, with the *u*‐axis and *v*‐axis defined according to the projection directions of the *p*‐waves and *s*‐waves electric fields. The top view (view 2) along the ‐*Z*‐axis and the rear view (view 4) along the ‐*Y*‐axis are set. (b) Reflection coefficients for copolarized and cross‐polarized waves in TM modes. *r*_pp_(60°) is smaller than *r*_sp_(60°) across the broadband range. (c) Reflection coefficients for copolarized and cross‐polarized waves in TE mode. *r*_ss_(60°) and *r*_ps_(60°) remain close and low across the broadband range. Polarization rotation angle *ψ* and ellipticity angle *χ* in (d) TM mode and (e) TE mode. The *ψ* value indicating deviation of the polarized waves principal axis from the original axis ranges between −90° and 90°. The ellipticity angle *χ* ranges from −45° to 45°, where 0° indicates linear polarization and ± 45° signifies circular polarization. (f) In view 2, surface induced currents in the gold films excited by incident waves in TM and TE modes at *λ* = 9.08 and 10.97 μm. The current directions on the G_1_ and G_2_ surfaces and on the gold substrate surface are labeled by red solid line arrows. The current is decomposed into *u*‐axis and *v*‐axis components. (g) In view 4, the spatial distribution of *Q*_diss_ for TM and TE modes at *λ* = 9.08 and 10.97 μm.

To accurately describe the reflected waves states, we introduce the polarization rotation angle *ψ* and ellipticity angle *χ* via the Stokes method to characterize the polarization states of the reflected waves (detailed calculations for *ψ* and *χ* are presented in Supporting Information S1: Section 5) [56, 57]. Note that in some cases, *ψ* may become zero during complete polarization conversion, which should be treated as *ψ* + 90°. Figure 4d illustrates the polarization states of the reflected waves. The *ψ* and *χ* values indicate that after polarization conversion, the reflected waves become elliptically polarized waves with the main axis rotating, inevitably reducing the *E*_*Z*_ electric field intensity of the original TM mode incident waves. When the incident waves are TE mode, the cross‐polarized and copolarized reflection coefficients are shown in Figure 4c. However, a low *r*_ps_(60°) does not imply that the metasurface has a low degree of polarization conversion. As shown in Figure 4f, at *λ* = 9.08 and 10.97 μm, the induced current intensity of the L‐shaped nanoantenna on the upper surface in the TE mode exceeds that in the TM mode. This current, combined with the current on the lower gold substrate, provides electric fields along the *X*‐axis and *Y*‐axis directions. The mutual interference enhancement between the electric fields from the inner surface of G_1_ and the gold substrate contributes to the *E*_*Z*_ component. The coupled electric field direction is deflected toward the *x*‐direction (TM mode) in the *xyz*, thereby exciting the Berreman mode of the multilayer WSMs and causing strong absorption. This is embodied in Figure 4g, where the *Q*_diss_ intensity of the TE mode at specific *λ* in the corresponding WSMs layer exceeds that of the TM mode. Furthermore, *Q*_diss_ is distributed along the WSMs layer and the inner surface of the gold film G_2_, demonstrating the generation of SPPs. Consequently, a significant portion of the waves with an electric field direction biased toward the TM mode is highly absorbed, resulting in reduced reflected cross‐polarized components. The *ψ* and *χ* in Figure 4e indicate that the major axis of the elliptically polarized state closely follows the TM mode electric field direction across most *λ*, thereby providing a substantial *E*_*Z*_ electric field component.

The electromagnetic characteristics of TM and TE mode incident waves propagating through the metasurface at *θ* = −60° and *φ* = 26° are presented in Figure 5. Figure 5a shows the incident waves (indicated by the red dashed line) interacting with the oblique WSMs‐gold unit, with observation view 2 and view 4 similarly configured. The reflection coefficients for cross‐polarized and copolarized TM modes are shown in Figure 5b, where *r*_sp_(−60°) and *r*_pp_(−60°) exhibit low values across a broad spectral range. The underlying mechanism is investigated using surface current and *Q*_diss_ distributions. Surface current distributions for TM mode at *λ* = 9.08 and 10.97 μm are depicted in Figure 5e. In view 2, the surface induced current in the L‐shaped nanoantenna provides the electric field in the *X*‐axis direction, whereas the current on the lower gold substrate provides the electric field in the *Y*‐axis direction. The current intensity at *λ* = 10.97 μm is higher than at *λ* = 9.08 μm, corresponding to *r*_sp_(9.08 μm,−60°) < *r*_sp_(10.97 μm, −60°) in Figure 5b. However, compared to Figure 4f, the current intensity is weaker, reducing the *E*_*Z*_ component weakened due to polarization conversion while maintaining the excitation intensity of the Berreman mode. Consequently, in Figure 5d, the *Q*_diss_ distribution of the TM mode under view 4 is observed in the corresponding WSMs layer. The incident waves generates SPPs between the multilayer WSMs and the inner surface of gold film G_2_, further enhancing absorption and thereby reducing reflection. Figure 5c shows the cross‐polarized and copolarized reflection coefficients for the TE mode. The high *r*_ps_(−60°) value indicates that most incident waves undergo polarization conversion but fail to enter the structure and be absorbed by the WSMs layers. Figure 5e also presents surface current distributions for the TE mode at *λ* = 9.08 and 10.97 μm. The current distributions in the L‐shaped nanoantenna and lower gold substrate under view 2 provide electric fields along the *X*‐ and *Y*‐axes directions, exhibiting stronger current intensities than those in the TM mode. Following the analytical logic from Figure 4, a high degree of polarization conversion should provide more *E*_*Z*_ components to excite the Berreman mode of the WSMs layers. However, in Figure 5d, the *Q*_diss_ of the TE mode incident waves in view 4 shows significantly weakened local intensity within the corresponding WSMs layers, failing to achieve high absorption. To explain this anomaly, the electric field distribution in Figure 5f reveals that the TM mode electric field energy is primarily concentrated within the WSMs and gold film G_2_. This indicates that the incident waves are highly absorbed after entering the WSMs layers. Conversely, the electric field energy of the TE mode is predominantly distributed on the outer surface slope of gold film G_1_. This occurs because the incident waves at −*θ* direction contacts a larger area of the slope. Consequently, due to the electromagnetic shielding effect of the gold, a significant portion of the incident waves undergoes polarization conversion on the gold surface and is reflected, preventing entry into the WSMs layers. Following the analysis of Figures 4 and 5, it is concluded that at *φ* = 26°, the TM mode exhibits high cross‐polarization reflection *r*_sp_(60°) and the TE mode exhibits low *r*_ps_(60°) at *θ* = 60°, whereas at *θ* = −60°, the TM mode exhibits high *r*_sp_(−60°) and the TE mode exhibits low *r*_ps_(−60°). Therefore, for incident waves with same polarization modes, the polarization conversion effect exhibits distinct asymmetry between +*θ* and −*θ*. This asymmetry facilitates the coupling to the magnetic WSMs, ultimately leading to the realization of nonreciprocal absorptivity/emissivity over a wide spectrum in both TM and TE modes.

**FIGURE 5:**
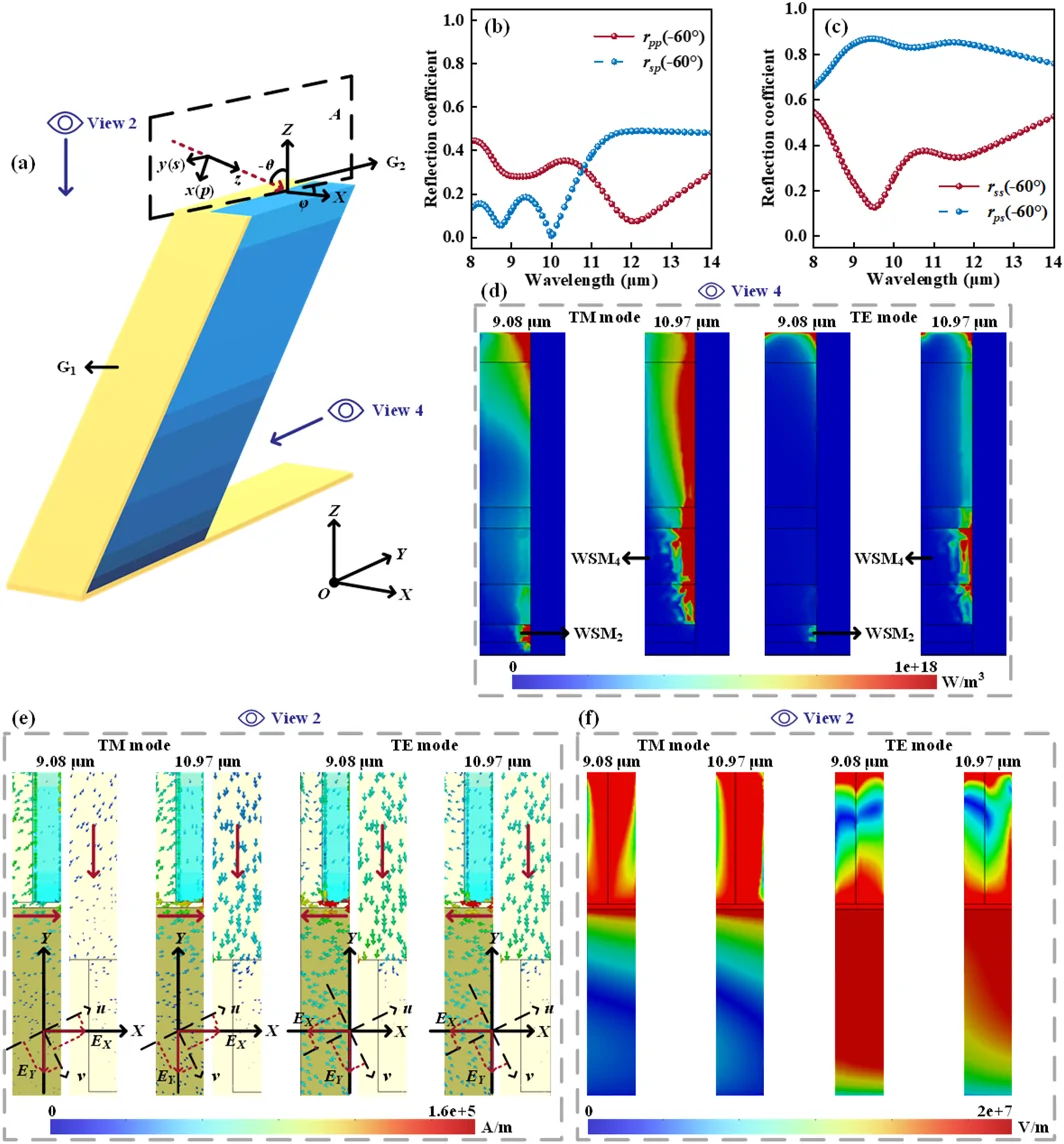
Performance analysis of the oblique WSMs‐gold unit at *θ* = −60° and *φ* = 26°. (a) Designed oblique WSMs‐gold unit. Within the incident plane at *φ*, the incident waves (red dashed line) strikes at *θ* = −60°. View 2 (top view along the −*Z*‐axis) and view 4 (rear view along the −*Y*‐axis) are set. (b) Reflection coefficients for copolarized and cross‐polarized in TM mode. *r*_pp_(−60°) and *r*_sp_(−60°) remain close and at low values across the broadband range. (c) Reflection coefficients for copolarized and cross‐polarized TE modes. *r*_ss_(−60°) exceeds *r*_ps_(−60°) across a broad bandwidth. (d) Spatial distribution of *Q*_diss_ for TM and TE modes at *λ* = 9.08 and 10.97 μm under view 4. (e) Surface induced currents in the gold layer excited by incident waves in TM and TE modes at *λ* = 9.08 and 10.97 μm under view 2. (f) Electric field energy distribution in TM and TE modes at *λ* = 9.08 and 10.97 μm under view 2.

Additionally, to further elucidate the physical origin of the nonreciprocity, we analyze the spectral response at limiting azimuth angles. As detailed in the Supporting Information S1: Section, at *φ* = 90°, where the geometric asymmetry is maximized (incident plane parallel to the tilt), the device surprisingly exhibits reciprocal behavior (*α*(*λ*,*θ*,*φ*) = *α*(*λ*,−*θ*,*φ*)) under TM and TE modes. When the *φ* = 0° (incident plane perpendicular to the tilt), the TE mode shows reciprocal behavior due to only *E*_*y*_ electric field components, which suppresses the excitation of magnetic coupling modes. This effectively rules out geometric scattering as the cause of nonreciprocity, attributing the effect strictly to magneto‐optical coupling which requires specific tensor interactions. Consequently, the artificial rabbit optimization algorithm is employed to identify the optimal angle of *φ* = 26°, which activate the magneto‐optical polarization conversion under both TM and TE modes to maximize the nonreciprocity intensity.

For TM and TE modes, the average emissivity across a broad spectral range (8–14 μm) is investigated. In Figure 6a,b, the emissivity curves exhibit significant asymmetry at opposite *θ*. We mark regions with average emissivity exceeding 0.7 using pentagram symbols to evaluate the metasurface thermal radiation performance. For TM mode at *θ* range from −83° to −47° and TE mode at *θ* range from 43° to 65°, *ē*_TM_(*λ*,*θ*,*φ*) and *ē*_TE_(*λ*,*θ*,*φ*) surpass 0.7. Conversely, emissivity remains relatively low in the opposite half‐plane, indicating the metasurface wide‐angle NTR characteristics. Further calculations reveal the average wavelength emissivity for TM and TE modes corresponding to the plane *A* after rotation the *φ* around the +*Z*‐axis as shown in Figure 6c,d. *φ* and *φ* + 180° correspond to incident waves at +*θ* and −*θ* within the same plane *A*. Triangular markers indicate the average emissivity at *φ* = 90° and 180°. Due to the momentum separation 2*b* of Weyl nodes along the +*Y*‐axis, the nonreciprocity between +*θ* and −*θ* incident waves gradually weakens until it disappears as *φ* approaches 90° or 180°. This occurs because the missing pair of electric field components oscillating perpendicularly to 2*b*, which leads to the nonexistence of NTR. For *φ* ≠ 90° and 270°, the emissivity at *φ* differs from that at *φ* = *φ*+ 180 °, demonstrating the nonreciprocity of the designed metasurface. Additionally, the *ē*_TM_(*λ*, *θ*, *φ*) and *ē*_TE_(*λ*, *θ*, *φ*) spectral lines exhibit near‐symmetrical distributions around *φ* = 90° and 270°, indicating that TM and TE modes can achieve +*θ* and −*θ* nonreciprocal emissivity within the first (*φ* = 0°–90°) and third quadrants (*φ* = 180°–270°). Simultaneously, the potential for achieving +*θ* and −*θ* broadband NTR in the second (*φ* = 90°–180°) and fourth quadrants (*φ* = 270°–360°) is also demonstrated. The counterclockwise rotation angle *γ* of the oblique WSMs‐gold unit around the +*X*‐axis for is varied from 0° to 90°. Although maintaining *φ* = 26°, Figure 6e,f show the average emissivity for TM and TE modes at *θ* = −60° and 60°, respectively. For the TM mode, within the structural inclination range of *γ* = 8°–55°, the incident waves at *θ* = −60° exhibit *ē*(*λ*,*θ*,*φ*) > 0.7. Conversely, for the TE mode, the incident waves at *θ* = 60° demonstrate *ē*(*λ*,*θ*,*φ*) > 0.7 within the *γ* = 27°–52°. As the *γ* decreases, the values of *ē*(*λ*,*θ*,*φ*) for the incident waves at +*θ* and −*θ* get closer and become smaller. This occurs because the slope effect of gold film G_1_ gradually disappears. At this point, all four sides of the multilayer WSMs become fully covered by gold films, leading to weakened polarization conversion asymmetry and enhanced electromagnetic shielding by the gold. Under strong reflection from the gold on the top surface and all four sides, the incident waves cannot penetrate into the WSMs interior to excite Berreman modes and intrinsic nonreciprocity. This corresponds to the reduced absorption and weakened nonreciprocity observed in Figure 6e,f. Furthermore, the Berreman mode triggered by ENZ WSMs not only possesses the advantages of wide‐angle NTR but also shows good robustness against thickness errors in metasurface processing. The discussion in Supporting Information S1: Section 7 demonstrates that for both TM mode and TE mode, under thickness variations of −15%–30% and −15%–25%, respectively, the nonreciprocity of the average emissivity of NTR is greater than 0.5.

**FIGURE 6:**
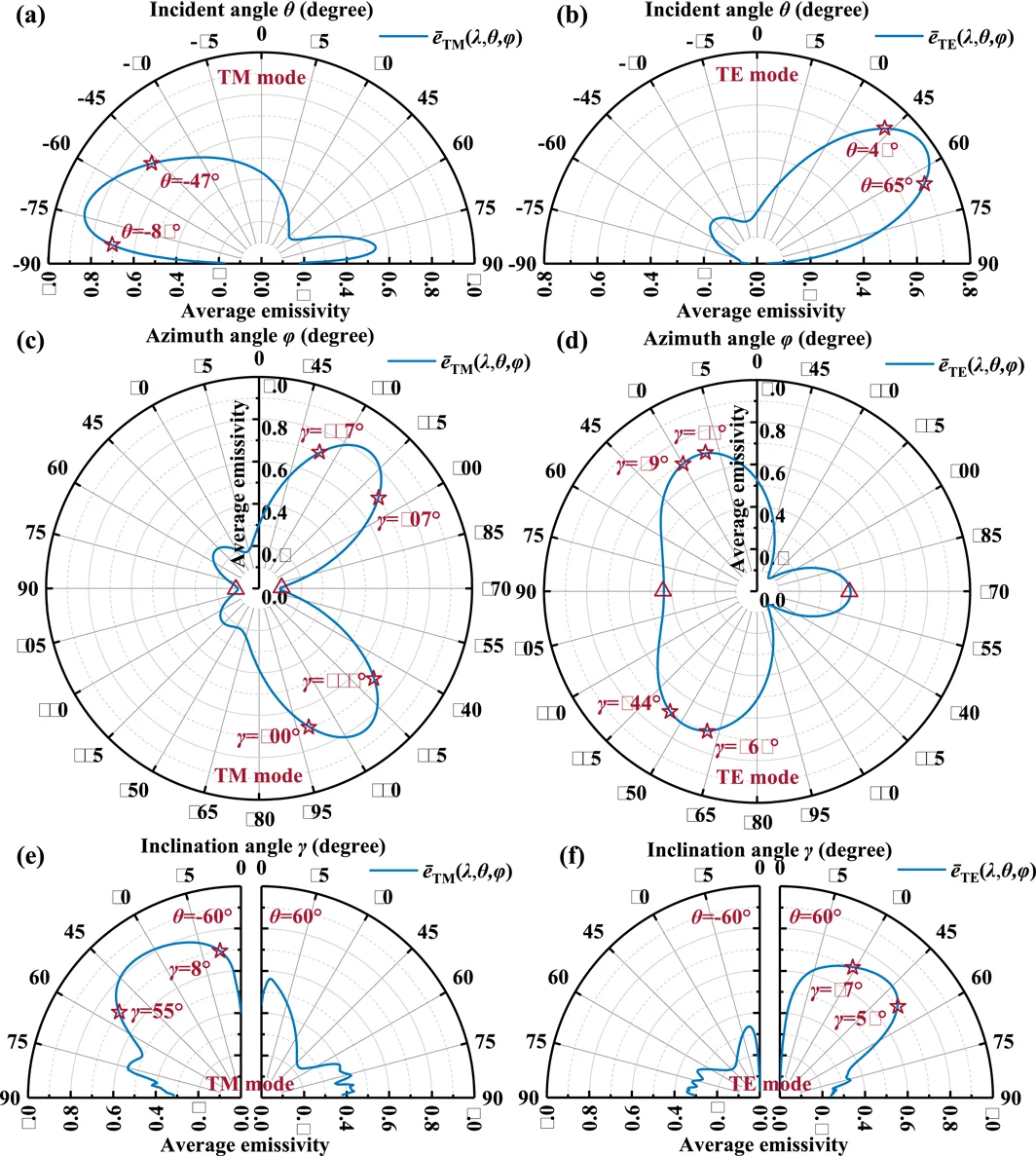
Analysis of the average emissivity as a function of incident angle *θ*, azimuth angle *φ*, and inclination angle *γ* for (a), (c), (e) TM mode and (b), (d), (f) TE mode. Red pentagrams mark regions where the average emissivity exceeds 0.7 across the 8–14 μm range. (a), (b): Polar coordinate distribution curves of the average emissivity for the metasurface under TM and TE modes as a function of the incident angle *θ* within −90° to 90°. (c), (d): Curves of the average emissivity of the metasurface as a function of the azimuth angle *φ* of plane *A* rotation, ranging from 0° to 360°, for both TM and TE modes. (e), (f): Distribution curves of the average emissivity for TM and TE modes at *θ* = 60° and −60° as the inclination angle *γ* of the oblique WSMs‐gold unit varies from 0° to 90°.

The preparation of WSMs can be achieved using solid‐state reaction techniques [58]. Recent experiments have begun to demonstrate the feasibility of growing WSMs films, such as the successful preparation of tantalum arsenide (TaAs) films using molecular beam epitaxy and pulsed laser deposition [59, 60]. These methods may also be applicable to other WSMs materials, such as Eu_2_IrO_8_. To realize the specific tilted multilayer geometry proposed in our design, glancing angle deposition [61] represents a viable fabrication strategy. This well‐established physical vapor deposition technique is widely capable of fabricating tilted columnar nanostructures. The gradient Fermi levels in WSMs can be tuned through doping experiments by controlling the type and concentration of dopants [62, 63, 64]. Regarding the metallic components, standard electron beam evaporation [65] is applicable for planar films. However, the critical L‐shaped gold cladding (sidewall plus top coverage) necessitates a more specific approach. It is anticipated that this asymmetric coverage could be achieved via a subsequent oblique‐angle shadow‐deposition process following the WSM growth. Utilizing the self‐shadowing effect of the pillars would allow for the precise coating of the targeted sidewalls, thereby creating the vertical plasmonic interfaces essential for the nonreciprocal mechanism. It should be noted that this work presents a general design approach for realizing NTR emitters with multipolarization and broad spectral range, achieved by combining ENZ materials with metasurface geometric construction techniques. Therefore, the WSMs used in this work can theoretically be replaced with other materials or structures exhibiting ENZ and intrinsic nonreciprocity, such as III–V semiconductors (e.g., InAs and InSb), magnetized ENZ metamaterials, and metasurfaces. According to the given geometric configuration, good multipolarization NTR performance can still be achieved.

Table 2 presents a comparison between this study and the latest research achievements. Up to now, only four works have investigated the realization of broadband NTR. We find that previous studies have only utilized the magneto‐optical effect of multilayer ENZ nonreciprocal materials and the Berreman mode to achieve highly asymmetric broadband absorption spectra (*α*(*λ*,*θ*,*φ*)≠*α*(*λ*,−*θ*,*φ*)) for TM‐polarized incident waves. However, due to the lack of magneto‐optical coupling terms in the TE mode, the Kirchhoff's radiation law still holds (*α*(*λ*,*θ*,*φ*) = *α*(*λ*,−*θ*,*φ*)). The method proposed in this work, which combines multilayer ENZ WSMs with metasurface geometric construction, can achieve broadband NTR from 8 to 14 μm simultaneously in both TM and TE modes, with excellent nonreciprocity *η*. This solves the bottleneck problem of being limited to TM polarization mode due to the inherent material properties and enables the utilization of radiative energy across multipolarization channels (albeit spatially separated), providing a radiator design strategy for the multipolarization utilization of radiative energy.

**TABLE 2:** Comparison of broadband NTR based on ENZ nonreciprocal materials performance.

Refs.	Years	Principle	Mode	*λ* range (μm)	*η*
[35]	2024	ENZ InAs	TM	12–18	0.147
[37]	2023	ENZ InAs	TM	2.73–4.29	0.3
[38]	2024	ENZ WSMs	TM	12–18	0.7
[39]	2025	ENZ InSb	TM	10–13	0.804
Our work	2025	ENZ WSMs + metasurface geometric	TM	8–14	0.696
			TE		0.732

It is worth noting that, as shown in Figure 6, the preferred emission directions for TE and TM modes are spatially separated (concentrated at +*θ* and −*θ*, respectively). Although this implies that a single‐direction collector cannot simultaneously harvest peak energy from both polarizations, this characteristic offers a unique advantage for polarization‐sorting energy harvesting. The proposed metasurface functions effectively as a thermal beam splitter, routing TE‐polarized thermal emission to the +*θ* spatial channel and TM‐polarized emission to the −*θ* channel. This spatial separation allows for the deployment of dual‐channel harvesters: collectors placed at +*θ* can specifically target TE energy, whereas those at −*θ*, target TM energy. This configuration potentially reduces cross‐polarization losses and simplifies the backend optical train compared to mixed‐polarization collection schemes.

## 4 Conclusion

In conclusion, by integrating a multilayer gradient ENZ WSMs with metasurface geometric construction techniques, we have for the first time proposed a metasurface emitter capable of achieving significant mid‐infrared NTR across a broad spectral range (8–14 μm) in both TM and TE modes. This breakthrough addresses the challenge faced in previous studies, where material inherent properties limited NTR to only the TM mode across a broad spectrum. This metasurface exhibits broadband low emission at +*θ* and high emission at −*θ* in the TM mode, whereas simultaneously achieving high emission at +*θ* and low emission at −*θ* in the TE mode. The high absorption across the broad spectrum is ensured by the Berreman mode excited by the electric field component *E*_*Z*_ of the incident waves within the multilayer WSMs featuring gradient ENZ variations. The broadband NTR is achieved through a two‐step process: (i) the structural anisotropy (geometric inclination and gold distribution) facilitates polarization conversion and (ii) the intrinsic magnetic properties of WSMs break Lorentz reciprocity to realize nonreciprocal absorption/emission. For the TM mode, high polarization conversion prevents the +*θ* incident waves from exciting the WSMs Berreman mode and being absorbed. Conversely, low polarization conversion at −*θ* allows coupling with the WSMs, exciting SPPs at the metal/dielectric interface and enhancing absorption. For the TE mode, high polarization conversion at +*θ* introduces a substantial *E*_*Z*_ component, exciting both the Berreman mode and SPPs for absorption. Although −*θ* incidence also exhibits high polarization conversion, it is largely reflected due to the electromagnetic shielding effect of the large‐area gold slope surface, significantly reducing absorption. Additionally, we investigate the influences of *θ*, *φ*, and *γ* on the broadband NTR performance. The stable average wavelength emissivity confirms the robustness of this metasurface NTR performance to incident waves directionality. The findings of this work will advance the development of broadband NTR emitters under multipolarization conditions, enhance the efficiency of radiative cooling and photon energy conversion/harvesting, and can be extended to the research and application of broadband NTR in other magnetized metals, III–V semiconductors, magnetized ENZ metamaterials, and metasurfaces.

## Author Contributions

**Junyang Sui:** writing – original draft, software, methodology, investigation, formal analysis, data curation, conceptualization. **Siyuan Liao:** writing – review and editing, methodology, conceptualization. **Haifeng Zhang:** writing – review and editing, supervision, methodology, funding acquisition, conceptualization.

## Conflicts of Interest

The authors declare no conflicts of interest.

## Supporting information


Supporting Information S1

## Data Availability

The datasets generated and/or analyzed during the current study are available from the corresponding author upon reasonable request.
