# Supplementary material for: Simultaneous Broadband Nonreciprocal Thermal Emission for Transverse Magnetic and Electric Polarizations
Source: Nanophotonics. 2026 Mar 20;15(6):e70046. doi: 10.1002/nap2.70046 (PMC13642162; doi:10.1002/nap2.70046)
Supplement: Supplementary file 1 [file NAP2-15-e70046-s001.docx]

Supporting Information

Junyang Sui, Siyuan Liao, and Haifeng Zhang*^[1]^

Simultaneous Broadband Nonreciprocal Thermal Emission for Transverse Magnetic and Electric Polarizations

1 **Artificial rabbits optimization: A new bio-inspired meta-heuristic algorithm for solving engineering optimization problems**

The Artificial Rabbit Optimization (ARO) algorithm is a nature‑inspired metaheuristic based on the survival strategies of rabbits, designed to address engineering optimization problems. It is particularly well suited for continuous optimization tasks and offers significant advantages when dealing with broadband, multimodal, non‑convex, and nonlinear problems.

The arrangement of the structural components in Figure 1(a) is maintained in the following sequence along the +*z*-axis. By varying the thickness of each dielectric layer (WSMs heights *h*_1_~*h*_7_, *w*_G2_, *w*_WSMs_, *l*_G2_), the Fermi levels (*E_F_*_1_~*E_F_*_7_), the rotation angle *γ*, incident angle *θ*, and azimuth angle *φ*, we generated a dataset with different designs *X*. In the metasurface design task of this work, the rabbit population size is *n_pop_*=50, the problem dimension is *d*’=20 (representing the optimized structural parameters), and the number of iterations is *It*=1000. The optimization target is set for the responses to satisfy *α*_TM_(*λ*,*θ*,*φ*)<0.3, *e*_TM_(*λ*,*θ*,*φ*)>0.85, *α*_TE_(*λ*,*θ*,*φ*)>0.85 and *e*_TE_(*λ*,*θ*,*φ*)<0.3. Throughout the optimization process, the Transmission matrix method is used to compute *α*(*λ*,*θ*,*φ*) and *e*(*λ*,*θ*,*φ*) for the metasurface within the *λ* range of 8 μm to 14 μm, with a step size of 10 nm.

The core principles of the ARO algorithm can be summarized in two main aspects [1,2,3].

(1) Detour Foraging: This strategy simulates rabbits detouring to graze near other burrows in order to avoid predator detection. By foraging around the edges in this manner, the algorithm enhances its exploration capabilities, thereby increasing the likelihood of uncovering superior solutions within the search space.

$\begin{matrix} \overrightarrow{v_{i}}(t+1) =\overrightarrow{x_{j}} (t) + R\cdot(\overrightarrow{x_{i}}(t)-\overrightarrow{x_{j}}(t)) + \text{round}(0.5\cdot(0.05+r_{1})) \cdot n_{1}, \\ i,j=1,\ldots,n_{pop}, j\neq i \end{matrix}$(S1)

$R=L\cdot c$ (S2)

$L=(e-e^{(\frac{t-1}{T})^{2}}) \sin(2\pi r_{2})$ (S3)

(S4)

$g=\text{randperm}(d')$ (S5)

$n_{1}\sim N(0,1)$ (S6)

Here, $\overrightarrow{v_{i}}(t+1)$denotes the candidate position of the *i*-th rabbit at iteration *t*+1 and $\overrightarrow{x_{i}}(t)$is current position at iteration *t*. *n_pop_* is the population size, *d*’ is the problem dimension, and *T* is the maximum number of iterations. is the ceiling operator, while round rounds to the nearest integer. randperm(*d*’) returns a random permutation of the integers 1 through *d*’. *r*_1_, *r*_2_, and *r*_3_ are independent samples drawn uniformly from (0,1). *L* is the running length, controlling the search step size during foraging. Finally, *n*_1_ is a random variable sampled from the standard normal distribution.

(2) Random Hiding: When foraging, rabbits randomly shelter in one of several nearby burrows to lower predation risk. In ARO, this is modeled by randomly selecting candidate solutions each iteration to enhance diversity and global exploration.

 (S7)

$H = \frac{T-t+1}{T} \cdot r_{4}$ (S8)

$n_{2}\sim N(0,1)$ (S9)

$g(k)=\left\{\begin{matrix} 1, & k=j, \\ 0, & \text{else}, \end{matrix}\right. k=1,2,\ldots,d'$ (S10)

In each dimension, *d*’ burrows are generated around a rabbit’s current position. The parameter *H* (the hiding factor) decreases linearly from 1 to 1/*T* over the course of the iterations under stochastic perturbations. As a result, at early iterations the burrows are created within a relatively large neighborhood of each rabbit, and this neighborhood gradually contracts as the iteration count increases.

As noted above, rabbits are frequently pursued and attacked by predators. To survive, they must seek out a safe refuge. Consequently, each rabbit randomly selects one of the available burrows in which to hide and thus avoid capture. To mathematically model this stochastic hiding strategy, the following formulations are proposed:

$\vec{v}_{i}(t+1)=\vec{x}_{i}(t)+R (r_{4} \vec{b}_{i,r}(t) - \vec{x}_{i}(t)), i=1,\ldots,n$ (S11)

 (S12)

$\vec{b}_{i,r}(t) = \vec{x}_{i}(t) + H f_{r} \vec{x}_{i}(t)$ (S13)

Here, $\vec{b}_{i,r}(t)$ denotes the burrow randomly selected out of the *d* available for hiding. *r*_4_ and *r*_5_ are independent random numbers drawn from the interval (0,1). The *i*-th foraging agent (rabbit) attempts to update its position by selecting one of its *d*’ burrows at random. After executing either the evasion‐foraging or the random-hiding strategy, the rabbit’s position is updated as follows:

After applying either the detour‐foraging or the random‐hiding strategy, the rabbit’s position is updated as:

$\vec{x}_{i}(t+1)=\left\{\begin{matrix} \vec{x}_{i}(t), & f(\vec{x}_{i}(t))\leq f(\vec{v}_{i}(t+1)) \\ \vec{v}_{i}(t+1), & f(\vec{x}_{i}(t))>f(\vec{v}_{i}(t+1)) \end{matrix}\right.$ (S14)

This formulation indicates that if the fitness at the candidate position exceeds that at the current position, the rabbit abandons its current location and updates to the candidate position produced by either Equation S1 or Equation S3.

In the ARO algorithm, rabbits predominantly perform detour foraging during the early iterations and switch to random hiding in the later stages. This search behavior is governed by each rabbit’s energy, which decays over time. To model the transition from exploration to exploitation, an energy factor is introduced. In ARO, this energy factor is defined as follows:

$A(t) = 4 (1-\frac{t}{T}) \ln\frac{1}{r}$ (S15)

Here, *r* is a random number drawn uniformly from the interval (0,1). In the ARO algorithm, when the energy factor *A*(*t*)>1, rabbits are in the exploration phase and tend to forage by detouring into regions occupied by other rabbits, yielding the detour foraging behavior. Conversely, when *A*(*t*)≤1, rabbits enter the exploitation phase and preferentially dig and hide within their own burrows, leading to the “random hiding” behavior. By monitoring the value of *A*(*t*), ARO dynamically switches between exploration (*A*(*t*)>1) and exploitation (*A*(*t*)≤1).

The pseudocode of ARO is described in Figure S1, and the flowchart for the ARO is presented in Figure S2.

**Figure S1:** Pseudocode of ARO algorithm.

**Figure S2:** The flowchart of the ARO.

2 **Validity of the Zeroth-Order Approximation and Diffraction Analysis**

**Figure S3:** Analysis of diffraction order contributions. Calculated reflectivity of the zeroth-order specular mode (*R*_(0,0)_) and higher-order diffraction harmonics (*R*_(0,1)_, *R*_(0,-1)_, *R*_(1,0)_ and *R*_(-1,0)_). The waves in TM mode incident at the angle (a) +60°, and (b) -60°. The waves in TE mode incident at the angle (a) +60°, and (b) -60°. The insets provide zoomed-in views of the higher-order components, confirming that their contributions are negligible (<0.002) across the entire 8~14 μm operating bandwidth.

In the main text, the absorptivity is calculated using the relation *α*(*λ*,*θ*,*φ*)=1-*R*(*λ*,*θ*,*φ*), where *R*(*λ*,*θ*,*φ*) refers to the specular reflectivity. As pointed out in recent theoretical works regarding universal modal radiation laws [4,5], this relation holds strictly only when energy dissipation into higher-order diffraction channels is negligible. Although our structure has a periodicity *P_y_*≈4.3 μm that is comparable to the shortest operating wavelength (*λ*≈8 μm), theoretically allowing the existence of grazing diffraction orders near the 8 μm boundary, we perform rigorous numerical simulations to quantify the energy distribution among different diffraction orders.

Figure S3 presents the calculated reflectivity for the zeroth-order specular mode (*R*_(0,0)_) alongside the first-order diffraction harmonics (*R*_(0,1)_, *R*_(0,-1)_, *R*_(1,0)_ and *R*_(-1,0)_) for both polarizations at *θ*=±60°. As observed in the plots, the optical response is overwhelmingly dominated by the zeroth-order specular reflection. The efficiencies of all higher-order diffraction channels remain negligible across the entire 8~14 μm band. As shown in the insets, the magnitude of these higher-order terms is consistently below 0.002, even near the theoretical diffraction cutoff at 8 μm. These results confirm that the metasurface effectively operates in the subwavelength regime, where higher-order diffraction is either evanescent or carries negligible energy. Consequently, the assumption *α*(*λ*,*θ*,*φ*)≈1-*R*_(0,0)_(*λ*,*θ*,*φ*) is rigorously valid for our device design.

3 **Nonreciprocal thermal radiation**

The formula of absorptivity and emissivity are derived in detail here. There are three objects in free space at the same temperature *T*, as shown in Figure S4. Two blackbodies BL_1_ and BL_2_, are located on either side of the heat source S and have far-field effects on it. Considering the radiated electromagnetic (EM) waves from the BL_1_ incident on the surface of S at *θ*, the waves from the BL_2_ are at -*θ*.

The thermal exchange between BL_1_ and S is put on the focus and four related key processes are marked in Figure S4. Supposing negligible energy transmitted through S, one can attain [6,7]:

$R(\theta)+\alpha(\theta)=1$ (S16)

where *R*(*θ*) indicates the reflectivity of the EM waves from BL_1_ and absorptivity *α*(*θ*) measures how much energy from the same direction is absorbed. For another, the energy flowing in the direction from S to BL_1_ consists of two components: the emission from S to BL_1_ (blue line), and the reflected emission from BL_2_ (green line). The EM waves from BL_2_ transmitted through the surface are also considered to approach zero. Provided the second law of thermodynamics, no net energy flows in and out of the blackbody BL_1_. It can be deduced [6,7]:

$R(-\theta)+e(\theta)=1$ (S17)

**Figure S4:** The three-body system concerns a heat source and two blackbodies at the same temperature *T*_0_. Four key processes are marked: process 1 (red line): incident EM waves from BL_1_ to S, process 2 (blue line): emission EM waves from S to BL_1_, process 3 (green line): reflected EM waves from BL_2_ to BL_1_, and process 4 (orange line): incident EM waves from BL_2_ to S.

The final derivation absorptivity *α*(*θ*) and emissivity *e*(*θ*) are as follows [6,7]:

$\alpha(\theta)=1-R(\theta)$ (S18)

$e(\theta)=1-R(-\theta)$ (S19)

For general materials, *R*(*θ*) and *R*(-*θ*) are equal, in which case *α*(*θ*) and *e*(*θ*) are numerically the same, and conform to the conventional Kirchhoff’s law. Yet, the nonreciprocal material such as InAs and Weyl semimetal, results in *R*(*θ*)≠*R*(-*θ*), thus bringing about *α*(*θ*)≠*e*(*θ*). As a result, the more generalized Kirchhoff’s law should be expressed as *α*(*θ*)=*e*(-*θ*) on the condition of the same incident wavelength. Although the conventional Kirchhoff’s law is broken, yet, it should be put on the emphasis that BL_1_, BL_2_, and S still follow the second law of thermodynamics exactly. Equation S18 and S19 have no conflict with the law throughout the whole derivation.

4 **The Design consideration of the WSMs layers ordering**

**Figure S5.** Spectral absorptivity and emissivity variation of the multilayer WSMs at *θ*=60° and *φ*=26°. The spherical lines represent the Fermi level of WSMs decreasing gradually from bottom to top, while the star lines represent the Fermi level increasing gradually from bottom to top, in both (a) TM mode and (b) TE mode.

For incident waves with an incident angle *θ*=60° and azimuth angle *φ*=26°, as shown in Figure S5(a), the absorptivity and emissivity of the TM mode remain high and uniformly distributed across a broad band of 8~14 μm due to the excitation of Berreman modes in the multilayer WSMs, where the Fermi levels progressively decreases from bottom to top (WSM_1_→WSM_7_) as listed in Table 1. In Figure S5(b), the absorptivity and emissivity of the TE mode for WSM_1_→WSM_7_ are influenced by the imaginary part of the intrinsic permittivity of the WSMs. This occurs because the WSMs layers with the lower Fermi level is positioned at the top of the structure. This layer exhibits weaker “metallic” properties, resulting in poorer shielding of the deeper WSMs layers. Consequently, waves energy propagates efficiently toward the lower WSMs layers. However, reversing the Fermi levels gradient, arranging the layers from bottom to top as WSM_7_ to WSM_1_, which yields an opposite spectral response. Both the absorptivity and emissivity for TM and TE modes in Figure S5(a) and (b) shift toward shorter wavelengths with reduced amplitudes. This occurs because the top WSMs layers possesses a high Fermi level, exhibiting stronger “metallic” behavior that effectively shields waves propagation toward the lower WSMs layers. Consequently, the Berreman modes at the ENZ wavelength points corresponding to the lower WSMs layers cannot be excited, leading to reduced absorptivity and emissivity at longer wavelengths.

5 **The calculation of the polarization rotation angle *ψ* and ellipticity angle *χ***

To describe accurately the reflected waves, we introduced Stokes method to determine the polarization state by using four parameters with the same physics dimension as [8,9]:

$S_{0}=r_{kk}^{2}+r_{jk}^{2}$ (S20)

$S_{1}=r_{kk}^{2}-r_{jk}^{2}$ (S21)

$S_{2}=2r_{kk}r_{jk}\cos(\delta)$ (S22)

$S_{3}=2r_{kk}r_{jk}\sin(\delta)$ (S23)

Here, *δ*=*φ_j_*-*φ_k_* denotes the phase difference between *r_jk_* and *r_kk_*. After polarization conversion, the waveform characteristics of the reflected wave are defined by the polarization rotation angle *ψ* and the ellipticity angle *χ* [8,9].

$\tan(2\psi)=S_{2}/S_{1}$ (S24)

$\sin(2\chi)=S_{3}/S_{0}$ (S25)

The polarization rotation angle *ψ* refers to the angle by which the plane of polarization of light rotates relative to a fixed reference frame. The range of *ψ* is typically from -180° to 180°, where *ψ*=0° indicates no rotation, and *ψ*=90° corresponds to a 90° rotation of the polarization plane. Note that in some cases, *ψ* may become zero during complete polarization conversion, which should be treated as *ψ*+90°. The ellipticity angle *χ* characterizes the shape of the polarization ellipse, quantifying the tilt of the ellipse's axes. The ellipticity angle *χ* ranges from -45° to 45°. When *χ*=0°, the polarization is linear (the ellipse degenerates into a straight line). When *χ*=45° and -45°, the polarizations are right-handed circular polarization and left-handed circular polarization respectively (the ellipse becomes a perfect circle), which indicates that the lengths of the major and minor axes of the ellipse are equal.

6 **Decoupling geometric asymmetry from magnetic nonreciprocity via azimuthal analysis**

To vigorously distinguish between geometric directional asymmetry (reciprocal, *α*(*λ*,*θ*,*φ*)≠*α*(*λ*,-*θ*,*φ*), but *α*(*λ*,*θ*,*φ*)=*e*(*λ*,*θ*,*φ*)) and true magnetic nonreciprocity (nonreciprocal, *α*(*λ*,*θ*,*φ*)≠*e*(*λ*,*θ*,*φ*)), and to justify the optimization of the azimuth angle *φ*, we perform control simulations at the limiting angles of φ=0° and φ=90°.

As shown in Figures S6(c) and (d), we investigate the optical response at *φ*=90°. In this configuration, the plane of incidence (*y*-*z* plane) is parallel to the structural tilt direction (*y*-axis). This orientation represents the scenario of maximum geometric asymmetry. For positive incidence (+*θ*) and negative incidence (-*θ*), the light interacts with the “front” versus the “back” of the tilted nanopillars, creating the largest possible contrast in geometric projection and shadowing effects. Despite this maximized geometric asymmetry, the absorptivity spectra at ±*θ* for both TM and TE polarizations exhibit negligible splitting (*α*(*λ*,*θ*,*φ*)=*α*(*λ*,-*θ*,*φ*), satisfying Kirchhoff’s law). The absence of nonreciprocity arises because, at *φ*=90°, the electric field components of the TM wave (*E_y_*, *E_z_*) and the TE wave (*E_x_*) lack the necessary spatial overlap with the off-diagonal terms of the Weyl semimetal permittivity tensor to induce magneto-optical coupling. This counter-intuitive resul, where the strongest geometric asymmetry yields no nonreciprocity, definitively proves that geometric scattering alone cannot induce the violation of Kirchhoff’s law observed in our device.

Similarly, at *φ*=0° (incident plane perpendicular to tilt), the TE mode with only *E_y_* component (Figure S6(b)) shows reciprocal behavior (*α*(*λ*,*θ*,*φ*)≈*α*(*λ*,-*θ*,*φ*)), which suppresses the excitation of magnetic coupling modes. However, the TM mode (Figure S6(a)) can induce the magneto-optical coupling to generate nonreciprocity due to the *E_x_* and *E_z_* components.

Since both limiting angles (*φ*=0° and *φ*=90°) suppress the nonreciprocal effect either due to symmetry or lack of tensor coupling, rotating the azimuth to an intermediate angle is physically necessary. The specific angle *φ*=26° is identified via the ARO algorithm. This configuration effectively activate the magneto-optical polarization conversion under both TM and TE modes while maximizing the nonreciprocity intensity.

**Figure S6.** Calculated spectral absorptivity *α* at incidence angles of *θ*=60° (solid curves) and *θ*=-60° (dashed curves) for limiting azimuthal angles *φ*. Spectra at *φ*=0° for (a) TM and (b) TE polarizations. Spectra at *φ*=90° for (a) TM and (b) TE polarizations.

7 **Discussion on structural robustness against fabrication errors**

**Figure S7.** Sensitivity analysis of the broadband nonreciprocal performance to fabrication deviations. The curve represents the spectral average nonreciprocity intensity (|*ē*(θ)-*ē*(-θ)|) calculated over the 8~14 μm wavelength range as a function of the variation (Δ*d*) in the total height (*h*_1_~*h*_7_)of the 7-layer gradient WSMs stack, under the (a) TM mode, and (b) TE mode.

To validate the experimental feasibility, we investigate the sensitivity of the broadband performance to fabrication errors, specifically focusing on the height deviations of the WSMs layers. Figure S7 illustrates the variation of the spectral average nonreciprocity intensity (|*ē*(θ)-*ē*(-θ)|) across the 8~14 μm atmospheric window under random height disorders. Here, we set the height of WSMs layer as *h*_1~7_+Δ*d***h*_1~7_. The results demonstrate significant robustness. Under TM mode, the average nonreciprocity remains above 0.5 for height variations ranging from -15% to 30%. Similarly, the average nonreciprocity exceeds 0.5 within a deviation range of -15% to 25% under the TE mode. This insensitivity to structural error is fundamentally linked to the physical mechanism of the Berreman mode supported by the ENZ WSMs. Unlike geometric cavity resonances (e.g., Fabry-Perot modes) which require strict phase matching conditions dependent on precise cavity lengths, the Berreman mode is a radiative mode excited at the interface of ENZ materials. It inherently possesses a broader angular acceptance and lower sensitivity to the bulk thickness of the film stack, provided the gradient profile is approximately maintained. Consequently, the proposed emitter shows good tolerance to processing errors, making it suitable for large-scale fabrication.

**References**

[1] L. Wang, Q. Cao, Z. Zhang, S. Mirjalili, and W. Zhao, “Artificial Rabbits Optimization: A New Bio-Inspired Meta-Heuristic Algorithm for Solving Engineering Optimization Problems,” *Eng. Appl. Artif. Intell.*, vol. 114, p. 105082, 2022.
[2] R. Wang, S. Zhang, and B. **, “Improved Multi-Strategy Artificial Rabbits Optimization for Solving Global Optimization Problems,” *Sci. Rep.*, vol. 14, p. 18295, 2024.
[3] F. Anka, N. Agaoglu, S. Nematzadeh, M. Torkamanian-afshar, and F. S. Gharehchopogh, “Advances in Artificial Rabbits Optimization: A Comprehensive Review,” *Arch. Comput. Methods Eng.*, vol. 32, p. 2113–2148, 2025.
[4] D. A. B. Miller, L. Zhu, and S. Fan, “Universal modal radiation laws for all thermal emitters,” *Proc. Natl. Acad. Sci. U.S.A*., vol. 114, pp. 4336–4341, 2017.
[5] B. Zhao et al., “Nonreciprocal thermal emitters using metasurfaces with multiple diffraction channels,” *Phys. Rev. Appl*., vol. 16, p. 064001, 2021.
[6] J. Wu, H. Li, C. Fu, and X. Wu, “High Quality Factor Nonreciprocal Thermal Radiation in a Weyl Semimetal Film via the Strong Coupling between Tamm Plasmon and Defect Mode,” *Int. J. Therm. Sci.*, vol. 184, p. 107902, 2023.
[7] J. Wu, B. Wu, Z. Wang, and X. Wu, “Strong Nonreciprocal Thermal Radiation in Weyl Semimetal-Dielectric Multilayer Structure,” *Int. J. Therm. Sci.*, vol. 181, p. 107788, 2022.
[8] X. **g, X. Gui, P. Zhou, and Z. Hong, “Physical Explanation of Fabry–Pérot Cavity for Broadband Bilayer Metamaterials Polarization Converter,” *J. Lightwave Technol.*, vol. 36, p. 2322–2327, 2018.
[9] S. Wu, Z. Zhang, Y. Zhang, K. Zhang, L. Zhou, X. Zhang, and Y. Zhu, “Enhanced Rotation of the Polarization of a Light Beam Transmitted through a Silver Film with an Array of Perforated S-Shaped Holes,” *Phys. Rev. Lett.*, vol. 110, p. 207401, 2013.

1. ***Corresponding author:** **Haifeng Zhang**,College of Electronic and Optical Engineering and the College of Flexible Electronics (Future Technology), Nanjing University of Posts and Telecommunications, Nanjing, 210023, China, and State Key Laboratory of Millimeter Waves, Southeast University, Nanjing, 210096, China. e-mail: [hanlor@163.com or hanlor@njupt.edu.cn;](mailto:hanlor@163.com;) ORCID:0000-0002-9890-8345

   **Junyang Sui, Siyuan Liao:** College of Electronic and Optical Engineering and the College of Flexible Electronics (Future Technology), Nanjing University of Posts and Telecommunications, Nanjing, 210023, China.
